# Protective Effect of Apiaceous Vegetables against Total Western Diet- and Dextran Sulfate Sodium-Induced Colitis in Mice

**DOI:** 10.1016/j.tjnut.2026.101652

**Published:** 2026-06-07

**Authors:** Hee-Seop Lee, Laura E Ibarra, Bin Zuo, Jiangchao Zhao, Renee Fox, Quentin D Read, Manoj Gurung, Laxmi Yeruva, Chi Chen, Sabrina P Trudo

**Affiliations:** 1 School of Human Environmental Sciences, University of Arkansas, Fayetteville, AR, United States; 2 Division of Agriculture, Department of Animal Science, University of Arkansas, Fayetteville, AR, United States; 3 Arkansas Children’s Nutrition Center, USDA-Agricultural Research Service (ARS), Little Rock, AR, United States; 4 Southeast Area, USDA-ARS, Raleigh, NC, United States; 5 Microbiome and Metabolism Research Unit, SEA, USDA-ARS, Little Rock, AR, United States; 6 Department of Food Science and Nutrition, University of Minnesota, St. Paul, MN, United States

**Keywords:** celery, parsnip, apiaceae, inflammation, microbiome, DSS, colitis, colorectal cancer

## Abstract

**Background:**

Western-style dietary patterns are associated with colitis and colon cancer. Existing data indicate that intake of apiaceous (API) vegetables (e.g., celery, parsnip) may prevent inflammation-associated diseases.

**Objectives:**

We investigated in mice the effect of API supplementation to the total Western diet (TWD) against dextran sulfate sodium-induced colitis.

**Methods:**

Male C57BL/6J mice (8-wk-old; 15 per group) were fed TWD supplemented with 21% or 42% fresh API (wt/wt) and given 2% dextran sulfate sodium to induce colitis. Diet intake, body weight, and disease activity index were monitored. Serum was collected for cytokine/chemokine analysis and colonic tissues for histology and Western blot. Fecal samples were analyzed by 16S ribosomal ribonucleic acid gene sequencing and targeted/untargeted metabolomics. Phenotypic data were analyzed by analysis of variance with Tukey’s test. Microbiome data were centered log-ratio (CLR) transformed and analyzed using linear mixed models with adjusted pairwise comparisons.

**Results:**

API supplementation attenuated colitis phenotypes including weight loss (44% recovery; *P* < 0.001), colon shortening (57% recovery; *P* < 0.01), disease activity (59% lower; *P* < 0.001), cytokine/chemokine release (35%‒73% reductions; *P* < 0.05), and mucosal F4/80+ cells infiltration (80% reduction; *P* < 0.001). API also improved gut microbiota diversity and composition, increasing α diversity metrics (4.4%‒13.8%; *P* < 0.05), suppressing pathogenic bacteria (*Paraclostridium*, *Enterococcus*, *Eubacterium*; estimated CLR difference: –1.8 to –6.7; *P* < 0.001), and enriching beneficial bacteria (*Lachnospiraceae* and *Blautia*; estimated CLR difference: +1.6 to +3.0*; P* < 0.05). Furthermore, metabolomics indicated TWD consumption increased arachidonic acid and aliphatic aldehydes (by 109%‒510%; *P* < 0.001), and decreased short-chain and unsaturated fatty acids (by 30%‒91%; *P* < 0.001). API supplementation also mitigated TWD-derived functional metabolites (including bile acids; *P* < 0.05).

**Conclusions:**

These data indicate that API intake is beneficial for risk reduction of diseases associated with Western diets. However, further investigations are warranted to determine the mechanism behind these beneficial effects.

## Graphical abstract

**Figure:**
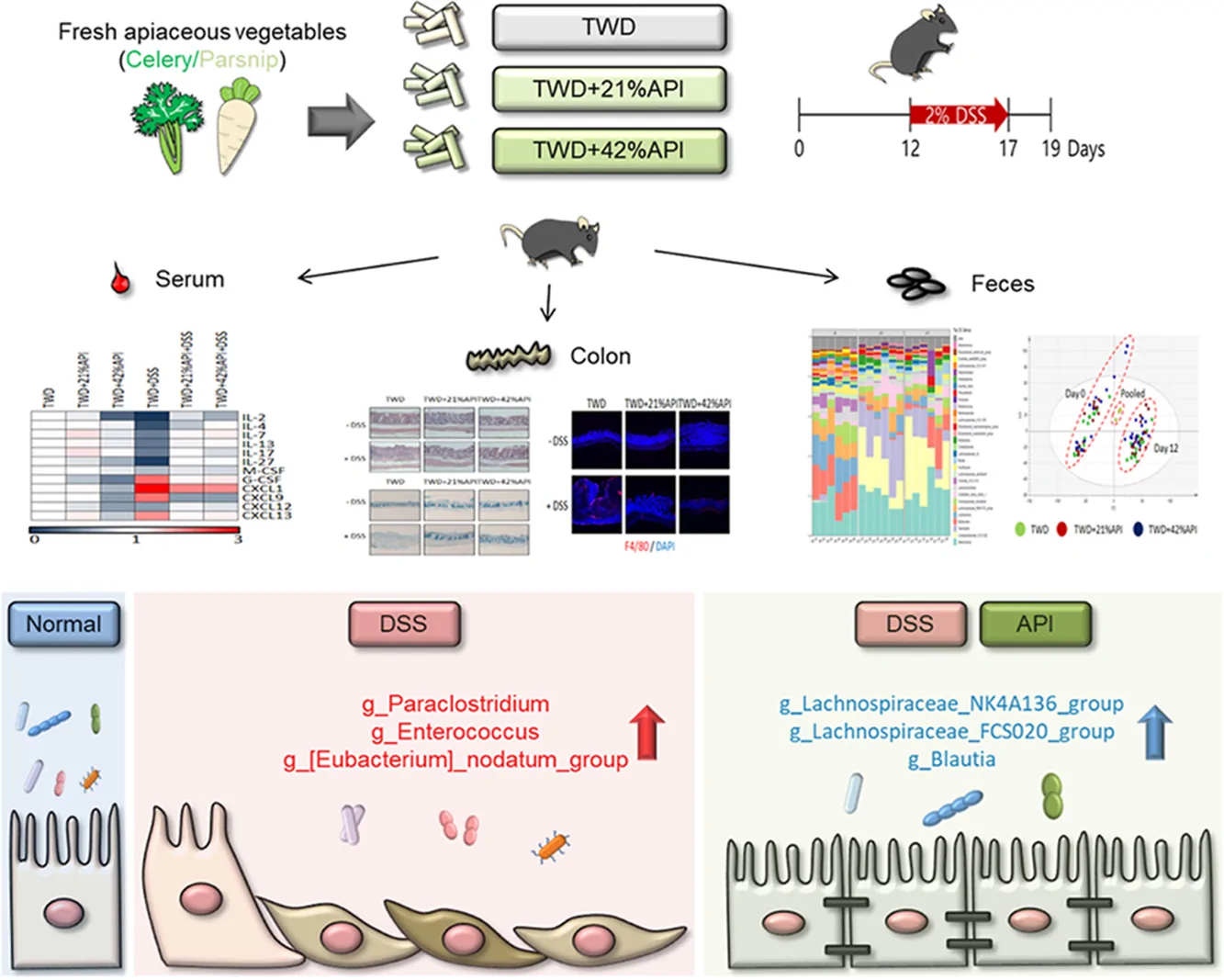

## Introduction

The incidence of inflammatory bowel diseases (IBD), including ulcerative colitis (UC) and Crohn’s disease (CD), continues to increase worldwide, especially in developing and Western countries [1]. Quality of life for IBD patients is severely compromised by symptoms such as diarrhea, abdominal cramps, hematochezia, weight loss, fever, fatigue, and anemia [2]. Chronic inflammation in IBD also increases colorectal cancer risk. Treatments include surgery, glucocorticoids, salicylic acid, and immunosuppressive agents, but with side effects [3]. The pathogenesis of IBD remains unclear, but is closely associated with genetic susceptibility, altered microbiota, and environmental factors that cause chronic inflammatory responses and impaired mucosal barrier function [4]. The gut microbiota may trigger IBD-initiating events, with composition in IBD patients clearly distinguishable from healthy individuals [5].

The Western-style diet is a major environmental risk factor for IBD and accompanying gut dysbiosis [[6], [7], [8]]. It is also considered a “putative element” for colorectal cancer, the third most common cancer in the United States with rising incidence among adults <50 y old [[9], [10], [11]]. A Western-style diet is characterized by highly processed and refined foods; high content of sugar, salt, and fat; and protein predominantly from red meat [12]. However, this dietary context is not consistently factored into the design of animal models of chronic disease. The Total Western Diet (TWD) is an experimental rodent diet reflecting the average macro- and micronutrient intakes of Americans [13]. Notably, consumption of TWD exacerbated the severity of colitis and incidence of inflammation-associated colorectal cancer, and altered the gut microbiome in mice [14,15].

Falcarinol and falcarindiol, the polyacetylenic oxylipins found in apiaceous (API) vegetables (e.g., celery, parsnip), have anti-inflammatory and anti-neoplastic effects in various models (as previously reviewed [16]) and specifically in the colons of rats [17,18]. Using a whole-food approach, pro-inflammatory cytokines were reduced in lung tissue of mice with acrolein-induced pulmonary injury but fed celery and parsnip [19]. Additionally, feeding celery and parsnip to rats and mice reduced colon cancer risk biomarkers after exposure to the foodborne colon carcinogen, 2-amino-1-methyl-6-phenylimidazo[4,5-b]pyridine [20,21]. Despite such consistency between phytochemical and whole-food data, a paucity of data remains regarding the whole-food effect of API vegetable intake on Western diet-associated diseases, such as IBD and gut microbial dysbiosis. Thus, the objective here was to assess whether API vegetables intake protects against experimental colitis in a dose-dependent manner in mice fed TWD, a more relevant background diet to mirror the Western-style dietary pattern and enhance translatability of murine models of diseases associated with this dietary pattern [15,22]. We also assessed alterations in gut microbial composition and function (i.e., metabolite profile).

## Methods

### Materials

Dextran sulfate sodium (DSS) was purchased from MP Biomedicals. Proteome profiler mouse cytokine array kit (#ARY006) was purchased from R&D Systems. Radioimmunoprecipitation assay, bicinchoninic acid assay kit, and enhanced chemiluminescence solution were purchased from Thermo Fisher Scientific. Primary antibodies (occludin and F4/80), horseradish peroxidase (HRP)-conjugated secondary antibody, and Alexa Fluor 594-conjugated secondary antibody were purchased from Cell Signaling Technology.

### Animals

Male C57BL/6J mice (7-wk-old; *n* = 90; Jackson Laboratory) were housed at the Central Laboratory Animal Facility (University of Arkansas, Fayetteville, AR) under controlled temperature (22°C ± 0.5°C), 50% humidity, and light conditions (12 h light-dark cycle) with free access to food and water. After 1-wk acclimation on a standard rodent unpurified diet (Teklad #8604, Inotiv; Supplemental Table 1), mice were fed experimental diets. Animal housing, handling, and experimental procedures were approved by the University of Arkansas Institutional Animal Care and Use Committee (protocol #22033).

### Diet and treatment

Diet ingredients for TWD were purchased from Dyets, Inc. As described previously [21], organically grown API vegetables (celery and parsnip) were purchased locally (Fayetteville, AR). Vegetables were washed, peeled (parsnip), ground by a Cuisinart Deluxe 11 11-Cup Food Processor (model DFP-11, Cuisinart), then mixed with TWD ingredients. After complete mixing, all diets were immediately divided into aliquots and placed into individual plastic bags for each study day, stored at ‒20°C, then thawed before feeding. The diet was replaced, and food intake was measured daily.

Using the standard =RAND() function in Microsoft Excel, mice were randomly assigned to 1 of 6 experimental groups (15/group) and housed as 7 or 8 mice per large cage (42.6 cm × 26.7 cm × 15.2 cm): group 1, TWD; group 2, TWD + 21% API (21% wet wt/wt; 10.5% per each vegetable); group 3, TWD + 42% API (42% wet wt/wt; 21% per each vegetable); group 4, TWD + DSS; group 5, TWD + 21% API + DSS; group 6, TWD + 42% API + DSS. As previously described, vegetable-supplemented diets were balanced for macronutrients and fiber using the USDA National Nutrient Database [23] (Supplemental Tables 2‒5). Due to the water content of vegetables and their influence on texture, diets were also matched for water. Supplementation of 21% API was equivalent to ∼128 g of vegetables per day (∼1 cup/d or 37 kcal; (∼256 g/d for 42%) for humans using allometric scaling based on energy intake as detailed previously [20]. Sample size per group was determined using an a priori sample size calculation (G∗Power v3.1.9.7), with an effect size of 0.5, an α level of 0.05, and statistical power of 0.95.

**FIGURE 1:**
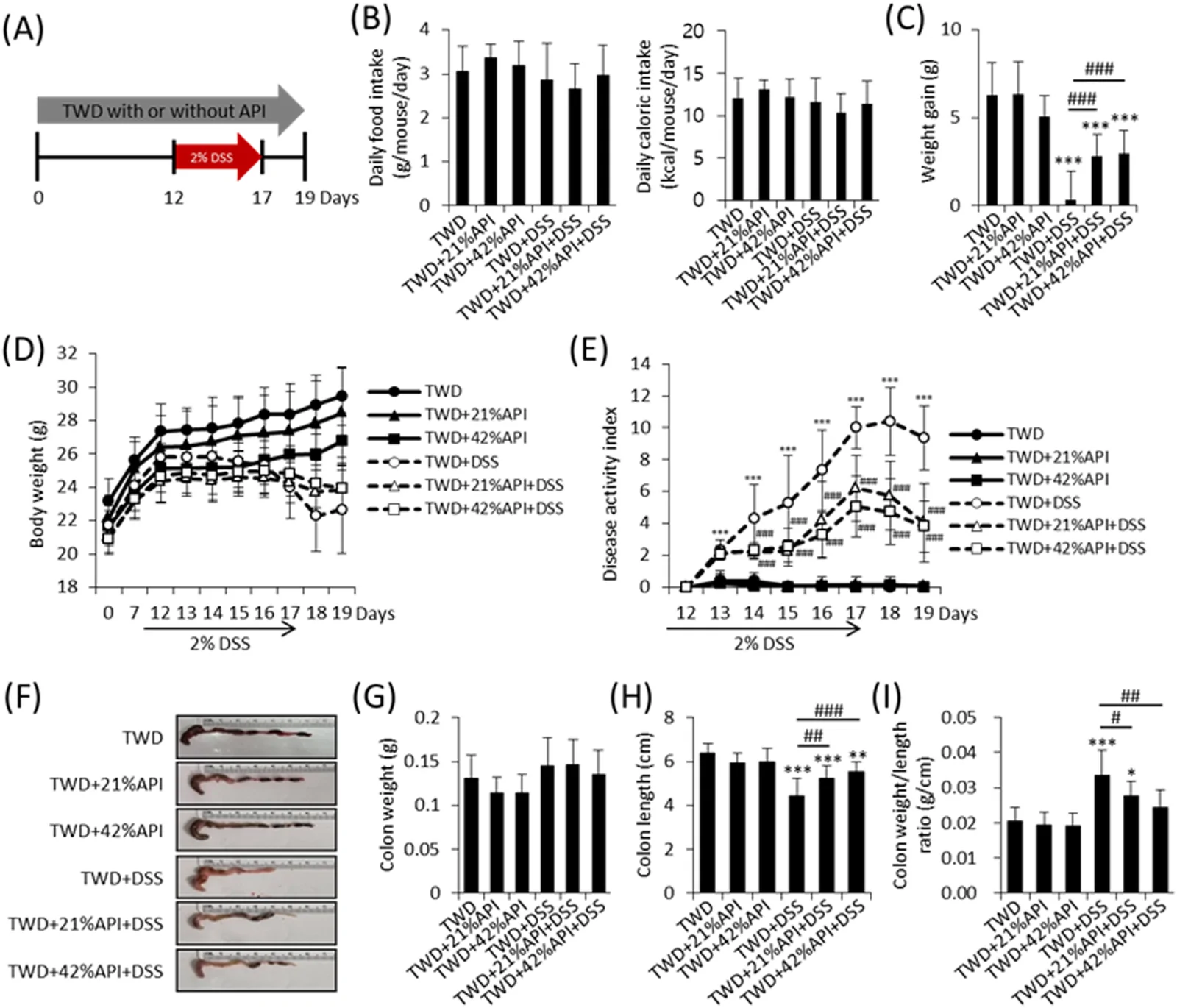
API vegetable supplementation attenuated DSS-induced colitis in mice. Mice were fed TWD with or without API for 19 d, and colitis was induced by DSS from day 12 to day 17 (A). Food and energy intake (B), net weight gain (C), body weight change (D), disease activity index (E), and colon weight and length (F‒I) were monitored during experimental periods. Data are presented as mean ± SD (*n* = 15). ∗*P* < 0.05, ∗∗*P* < 0.01, ∗∗∗*P* < 0.001 compared with TWD. #*P* < 0.05, ##*P* < 0.01, ###*P* < 0.001 compared with TWD + DSS. API, apiaceous; DSS, dextran sulfate sodium; TWD, total Western diet.

On day 12, mice in groups 4, 5, and 6 were administered 2% (wt/vol) DSS in drinking water for 5 d, then returned to regular drinking water (Figure 1A); mice in groups 1, 2, and 3 were provided regular drinking water throughout. Two days after withdrawal of DSS (day 19), mice were killed and blood collected by cardiac puncture. Colons were harvested, measured, flushed with phosphate-buffered saline, slit open longitudinally, divided into longitudinal sections, then either snap-frozen in liquid nitrogen for storage at ‒80°C for western blotting or formalin-fixed and paraffin-embedded for histologic evaluation.

### Disease activity index

In random order, the body weight of each mouse was recorded on day 0, day 7, and daily from day 12‒19. Disease activity index (DAI) was determined to evaluate colitis grade and extent [24]. Briefly, DAI scores were calculated by summing individual scores for: body weight loss (scored as: 0, <1%; 1, 1%‒5%; 2, 5%‒10%; 3, 10%‒15%; 4, >15%), stool consistency (scored as: 0, normal; 2, loose stools; 4, diarrhea), and blood in stool (scored as: 0, negative; 2, positive; 4, gross rectal bleeding).

### Tissue staining

Paraffin-embedded colon tissues were sectioned with a 4 *μ*m thickness. Deparaffinized tissue slides were stained with hematoxylin and eosin. The histological score was assessed by summing 3 parameters (scored as 0‒4 for each, with a maximum combined score of 12): surface epithelial loss, crypt destruction, and inflammatory cell infiltration into the mucosa [25]. The acidic mucus area was stained with Alcian blue (pH 2.5) for 30 min, then nuclei were counterstained with Fast Red solution for 5 min. Alcian blue-positive areas were measured by ImageJ software (v1.54i, NIH). For immunofluorescence staining, colon tissue sections were incubated with 10% normal goat serum for 1 h, then incubated with rabbit anti-F4/80 antibody (Cell Signaling Technology) overnight at 4°C. Tissue sections were washed with phosphate-buffered saline and incubated with Alexa Fluor 594-conjugated secondary antibody. Fluorescence images were taken under an inverted fluorescence microscope (VWR) equipped with a Moticam ProS5 camera (Motic), and the F4/80-positive area was measured by ImageJ.

### Western blotting

Colon tissues were lysed using radioimmunoprecipitation assay buffer, and protein concentration was measured by a bicinchoninic acid assay kit. Proteins were separated by sodium dodecyl sulfate polyacrylamide gel electrophoresis, transferred to nitrocellulose membranes, incubated with primary antibody, and then target proteins were incubated with HRP-conjugated secondary antibody and detected using enhanced chemiluminescence solution. Band intensity was quantified using ImageJ. Quantification of occludin was restricted to the full-length ∼65 kDa band given that the functional roles of lower-molecular-weight occludin species remain incompletely defined. For normalization, we quantified the predominant 42 kDa β-actin band.

### Cytokine array

Serum cytokines and chemokines were analyzed by a proteome profiler mouse cytokine array kit (R&D Systems). Briefly, membranes immobilized with cytokine/chemokine-capture antibodies were incubated with serum samples overnight at 8°C. After being washed, membranes were incubated with streptavidin-HRP for 2 h at room temperature. Chemiluminescence was detected by X-ray film. Cytokine/chemokine spots were analyzed by ImageJ software.

### DNA extraction and 16S rRNA gene sequencing

Fecal samples were collected sterilely from each mouse on day 0, day 12, and day 17, then stored at ‒80°C for future analysis. Genomic DNA was extracted from mice feces using the PowerLyzer PowerSoil DNA isolation kit (Qiagen) per the manufacturer’s instructions. Extracted DNA was quantified using a NanoDrop (Thermo Fisher Scientific) and diluted to 10 ng/*μ*L with DNase- and RNase-free water. Libraries were constructed according to published methods [26]. In brief, the V4 region of the bacterial 16S rRNA gene was amplified with universal primers (F: 5′-GTGCCAGCMGCCGCGGTAA-3′ and R: 5′-GGACTACHVGGGTWTCTAAT-3′). Amplicon size was verified by agarose gel electrophoresis. To clean up and normalize polymerase chain reaction products, the SequalPrep normalization plate kit (Invitrogen) was utilized. Normalized amplicons were pooled in equal volume. Their quality was measured with an Agilent Bioanalyzer 2100 (Agilent) and quantity measured by RT-PCR. For sequencing of the pooled amplicons, Illumina MiSeq 2 × 250 bp paired-end sequencing (MiSeq reagent kit v2) was performed. For quality control, each MiSeq analysis included negative controls for DNA extraction and polymerase chain reaction amplification, and a mock community standard (ZymoBIOMICS Microbial Community Standard, Zymo Research). Sequencing files from the current study are available in the Sequence Read Archive repository (SUB12284212).

### Microbiome analysis

Analysis was done at the genus and phylum levels; all data processing and analysis described hereafter were repeated twice, once for each taxonomic level. In both cases, taxa found in only 1 sample were excluded from further analysis.

First, a sparse partial least squares discriminant analysis (sPLS-DA) was fit separately to the microbiome abundance data for each of the 3 time points (days 0, 12, and 17). Data for each time point were transformed before model fitting by adding 1 to all counts, then applying the centered log-ratio (CLR) transformation. Treatment group (combination of diet treatment and DSS challenge) was treated as the dependent variable in the sPLS-DA. Taxa with near-zero variance, defined as having a ratio of 19:1 or greater between the most common and second most common value, were removed prior to model fitting. First, the optimal tuning parameters were selected for the sPLS-DA fit using 3-fold cross-validation with 100 repetitions. The 2 tuning parameters optimized were the number of *x*-variables to retain for each component and the number of components. For the phylum-level analysis, all combinations of 2, 3, 4, and 5 *x*-variables retained per component, and 1, 2, and 3 components were tested. For genus-level analysis, all combinations of 10, 20, 30, 40, 50, 60, and 70 *x*-variables and 1, 2, 3, 4, and 5 components were tested (Supplemental Table 6 shows the optimal values identified by cross-validation). After using the cross-validation performance to identify optimal tuning parameters, the model for each time point was refit using those parameters. Its prediction performance was determined using a second round of 3-fold cross-validation repeated 100 times. The centroid distance metric was the best performing compared to the maximum and Mahalanobis distance metrics. Predictions on the hold-out data points were made using the centroid distance metric, and the prediction error rate was calculated. Variable importance in the projection scores was computed for day 17 for each taxon to indicate which taxa differed the most across treatment groups.

Second, linear mixed models accounting for repeated measures within each individual over time were used to assess the effect of the treatments on the abundances of individual taxa at both the phylum and genus level. First, data for all time points combined were CLR transformed after adding to all counts. Next, a linear mixed model was fit to each taxon abundance. The fixed effects were diet treatment (categorical with 3 levels), DSS challenge (categorical with 2 levels), time point (categorical with 3 levels), and all 2-way and 3-way interactions. A random intercept was fit to each individual mouse. Marginal means and 95% confidence intervals were estimated for each combination of diet treatment and DSS challenge treatment for day 17. Within each DSS challenge, pairwise comparisons between each of the 3 diet means for each taxon were done using t-tests. *P* values were adjusted using the Benjamini-Hochberg method to maintain the false discovery rate at 0.05. There were 3 comparisons per taxon. At the phylum level, 6 phyla were compared within each DSS level, and at the genus level, 76 genera were compared; thus, adjustment was made for 18 comparisons at the phylum level and 228 comparisons at the genus level. A similar mixed model was fit to each of 6 metrics of α-diversity at the species level: observed richness, Chao1 richness estimator, Abundance-based Coverage Estimator (ACE), Shannon entropy, Simpson diversity, and Fisher’s α. Comparisons between diversity means between each pair of diets were done by pairwise *t*-tests within each DSS level. The *P* values for these comparisons, and the 95% confidence intervals around the means, were adjusted with the Sidak multiple-comparison adjustment for 3 comparisons each. For all mixed models, the Kenward-Roger method was used to approximate the denominator degrees of freedom. Data were analyzed using R software v4.3.1 [27]. Multivariate analyses used the package mixOmics v6.24.0 [28], and mixed-model analyses used the packages lme4 v1.1-34 [29], emmeans v1.8.9 [30], and multcomp v1.4-25 [31].

### Targeted metabolite analysis and untargeted metabolomics modeling

To explore possible mechanisms by which API-induced changes in microbiota-mediated protection against DSS-induced colitis, we conducted targeted and untargeted metabolomics analyses of day 12 fecal samples (collected immediately prior to DSS administration). For quantitative analysis of short-chain fatty acids (SCFA; acetic acid, propionic acid, butyric acid, valeric acid, and isovaleric acid), fecal samples were prepared by mixing with 50% aqueous acetonitrile in a 1:10 (wt/vol) ratio, then centrifuging at 18,000 × *g* for 10 min to obtain fecal-extract supernatants. Fecal extracts were first derivatized by 2-hydrazinoquinoline [32], separated by a Bridged Ethylene Hybrid (BEH) C18 column (Waters) in an Acquity ultraperformance liquid chromatography system (Waters), then detected in an Xevo-G2-S quadrupole time-of-flight mass spectrometer system (Waters). Conditions of liquid chromatography–mass spectrometry analysis, including mobile phase, and parameters of mass spectrometry detection have been described previously [33]. Mass chromatograms and mass spectral data were acquired and processed using the MassLynx software v4.2 (Waters) in centroided format. Individual compound concentrations were determined by calculating the ratio between the peak area of the compound and the peak area of the internal standard and fitting with a standard curve using the QuanLynx software v4.2 (Waters).

For untargeted metabolomics modeling, chromatographic and spectral data of samples were deconvoluted by MarkerLynx software (Waters) to generate a multivariate data matrix containing information on sample identity, ion identity (retention time and *m/z*), and ion abundance. The abundance of each ion was calculated by normalizing the single ion counts compared with the total ion counts in the entire chromatogram. The data matrix was then exported into SIMCA-P+ software (Umetrics) and transformed by Pareto scaling. Supervised partial least squares-discriminant analysis (PLS-DA) was used to model the fecal samples. Metabolite markers were identified by analyzing ions contributing to sample separation in PLS-DA models. The chemical identities of metabolite markers were determined by accurate mass measurement using elemental composition analysis, searching the Human Metabolome Database and the METLIN database, and by tandem mass spectrometry fragmentation and comparisons with authentic standards if available.

### Statistical analysis

Results, except for microbiome data, are represented as mean ± SD from the indicated number of mice in the figure legends (*N* = *X* per group); statistical difference was evaluated by 1-way analysis of variance followed by Tukey’s multiple-comparison test using GraphPad Prism 8.0 (GraphPad Software).

## Results

### API attenuated DSS-induced colitis

Daily food intake and caloric intake did not differ between the 6 groups (Figure 1B). During DSS challenge periods, mice in DSS-challenged groups lost weight due to diarrhea and rectal bleeding (Figure 1C and D). Net body weight gain was lower in TWD + DSS (0.4 ± 1.6 g, *P* < 0.001) than TWD (6.3 ± 1.9 g), which was improved in TWD + 21% API + DSS (2.8 ± 1.2 g, *P* < 0.001) and TWD + 42% API + DSS (3.0 ± 1.3 g, *P* < 0.001) compared to TWD + DSS. API supplementation attenuated the DSS-induced increase in DAI (Figure 1E). As expected, colon shortening was induced by DSS challenge, and colon weight/length ratio was increased by DSS; these improved with 21% and 42% API supplementation (Figure 1 F‒I).

### Cytokine/chemokine profiles were improved by API supplementation

**FIGURE 2:**
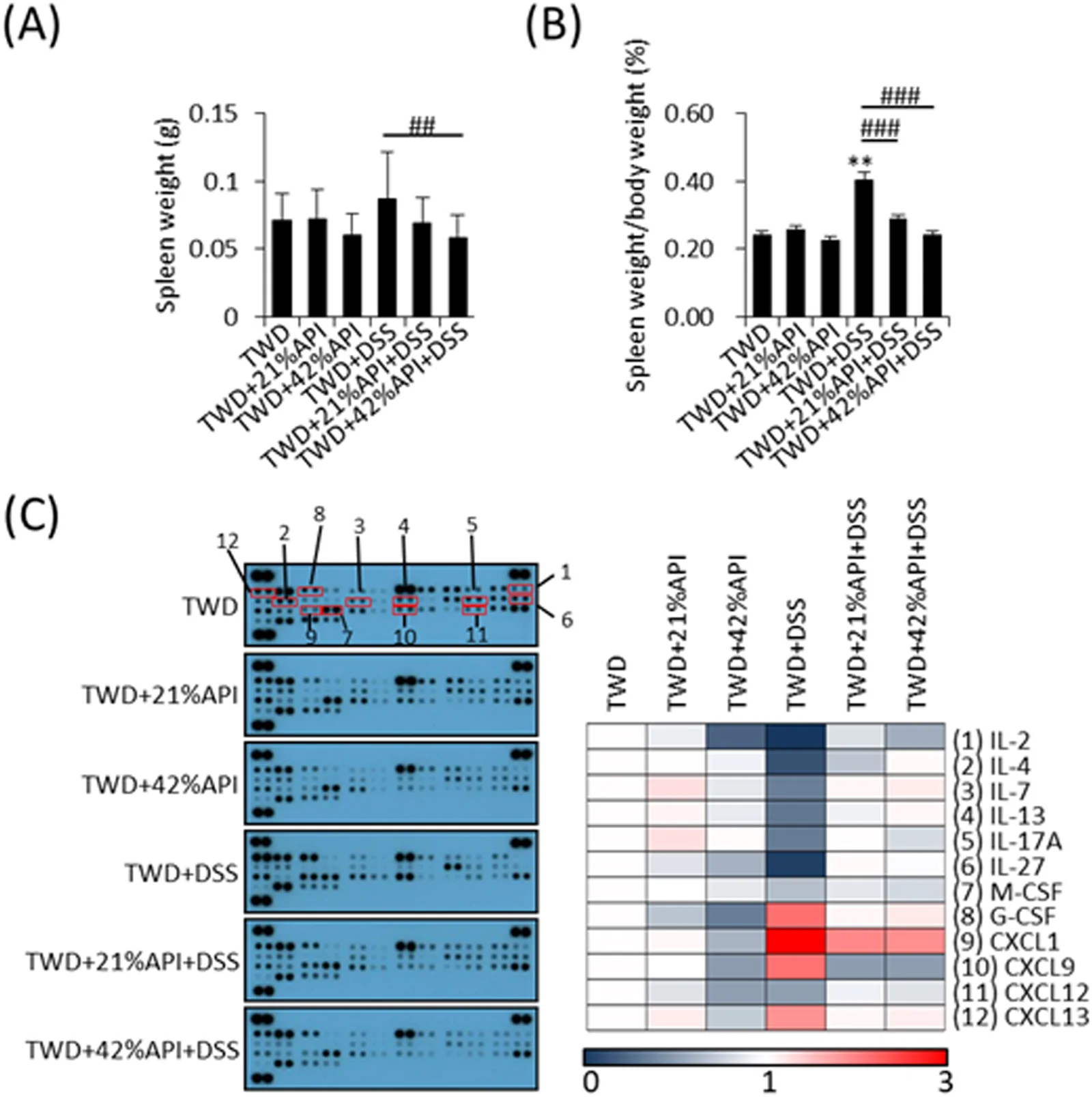
API vegetable supplementation attenuated DSS-induced spleen hypertrophy and serum cytokine and chemokine release in mice. (A) Spleen weight. (B) Spleen index (spleen weight/body weight ratio). (C) Cytokines and chemokines were measured as described in the Methods section. Representative blot images (left) are presented, and the relative levels of the selected ones are presented in a heat map (right). Darkening blue means decreased and darkening red means increased level relative to the TWD-only group. The information of each spot on the blot and the relative expression level of selected cytokines and chemokines can be found in Supplemental Figures 1 and 2 and Supplemental Table 3, respectively. Data are presented as mean ± SD (*n* = 15 for A, B; *n* = 4 for C). ∗∗*P* < 0.01, compared with TWD. ##*P* < 0.01, ###*P* < 0.001 compared with TWD + DSS. API, apiaceous; CXCL, C-X-C motif ligand; DSS, dextran sulfate sodium; G-CSF, granulocyte-colony stimulating factor; IL, interleukin; M-CSF, macrophage colony-stimulating factor; TWD, total Western diet.

Spleen weight in TWD + 42% API + DSS was lower than in TWD + DSS (Figure 2A). Spleen weight/body weight ratio (spleen index) was increased by DSS challenge (0.4% ± 0.2% in TWD + DSS compared to 0.2% ± 0.1% in TWD, *P* < 0.001) but was reversed in TWD + 21% API + DSS and TWD + 42% API + DSS (0.3% ± 0.1%, *P* < 0.05; 0.2% ± 0.1%, *P* < 0.001, respectively, Figure 2B). Serum cytokines IL-2, IL-4, IL-7, IL-13, IL-17A, and IL-27 were lower in TWD + DSS compared to TWD but were reversed by API supplementation (Supplemental Figures 1 and 2, Figure 2C, and Supplemental Table 7). The growth factors macrophage colony-stimulating factor and granulocyte-colony-stimulating factor were decreased and increased, respectively, in TWD + DSS compared to TWD; these changes were reversed in API supplemented groups challenged with DSS. C-X-C motif ligand (CXCL)-type chemokines (CXCL1, CXCL9, CXCL13) were increased, but CXCL12 was decreased in TWD + DSS; these were also reversed in TWD + 21% API + DSS and TWD + 42% API + DSS.

### API protected colonic mucosa against DSS-induced damage and F4/80-positive cell infiltration

**FIGURE 3:**
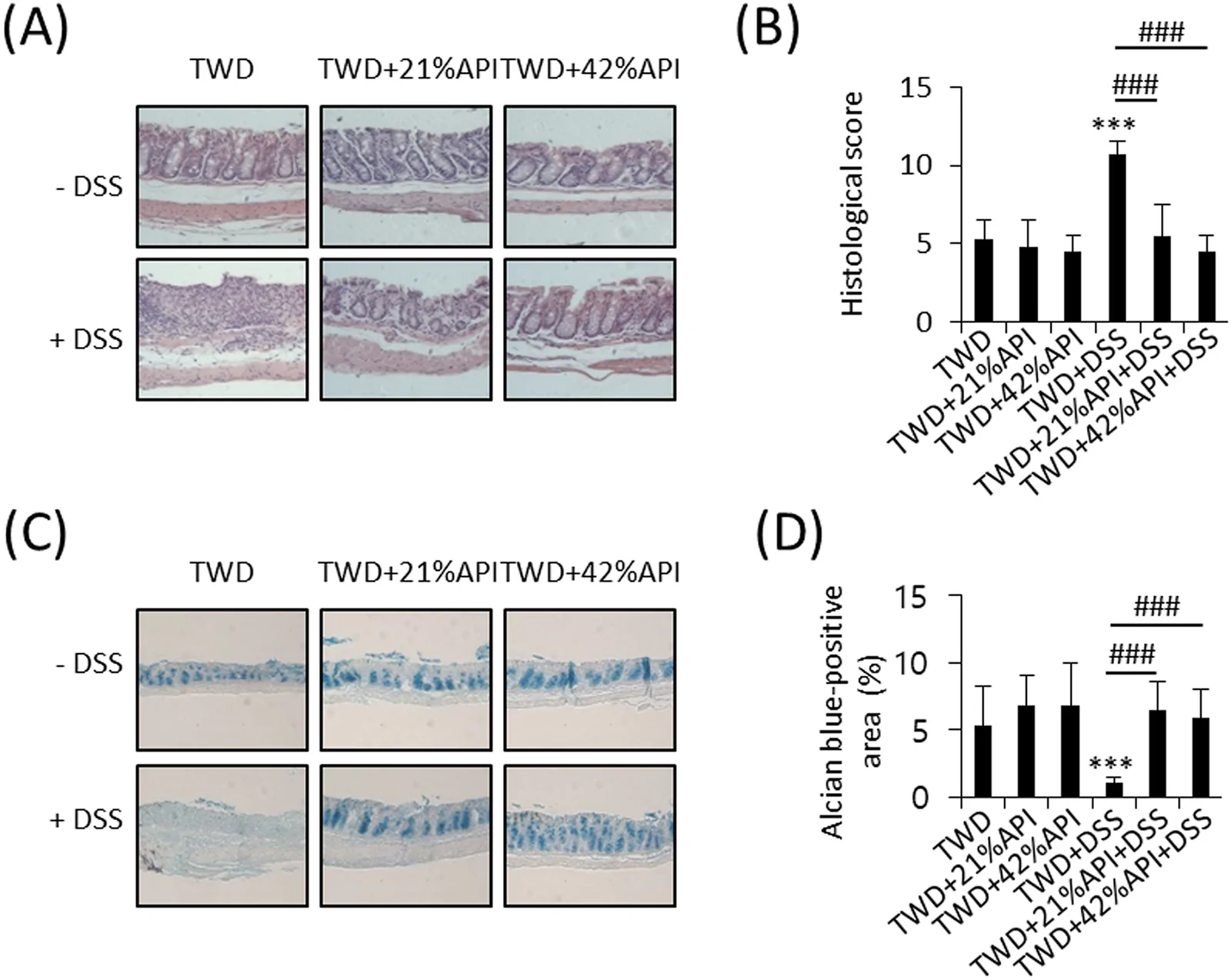
API vegetable supplementation mitigated colonic structural damage induced by DSS and protected the mucus layer. (A and B) Hematoxylin and eosin staining was performed to assess the histological score. (C and D) The mucus layer was stained with Alcian blue and quantified using ImageJ software. Data are presented as mean ± SD (*n* = 15). ∗∗∗*P* < 0.001 compared with TWD. ###*P* < 0.001 compared with TWD + DSS. API, apiaceous; DSS, dextran sulfate sodium; TWD, total Western diet.

**FIGURE 4:**
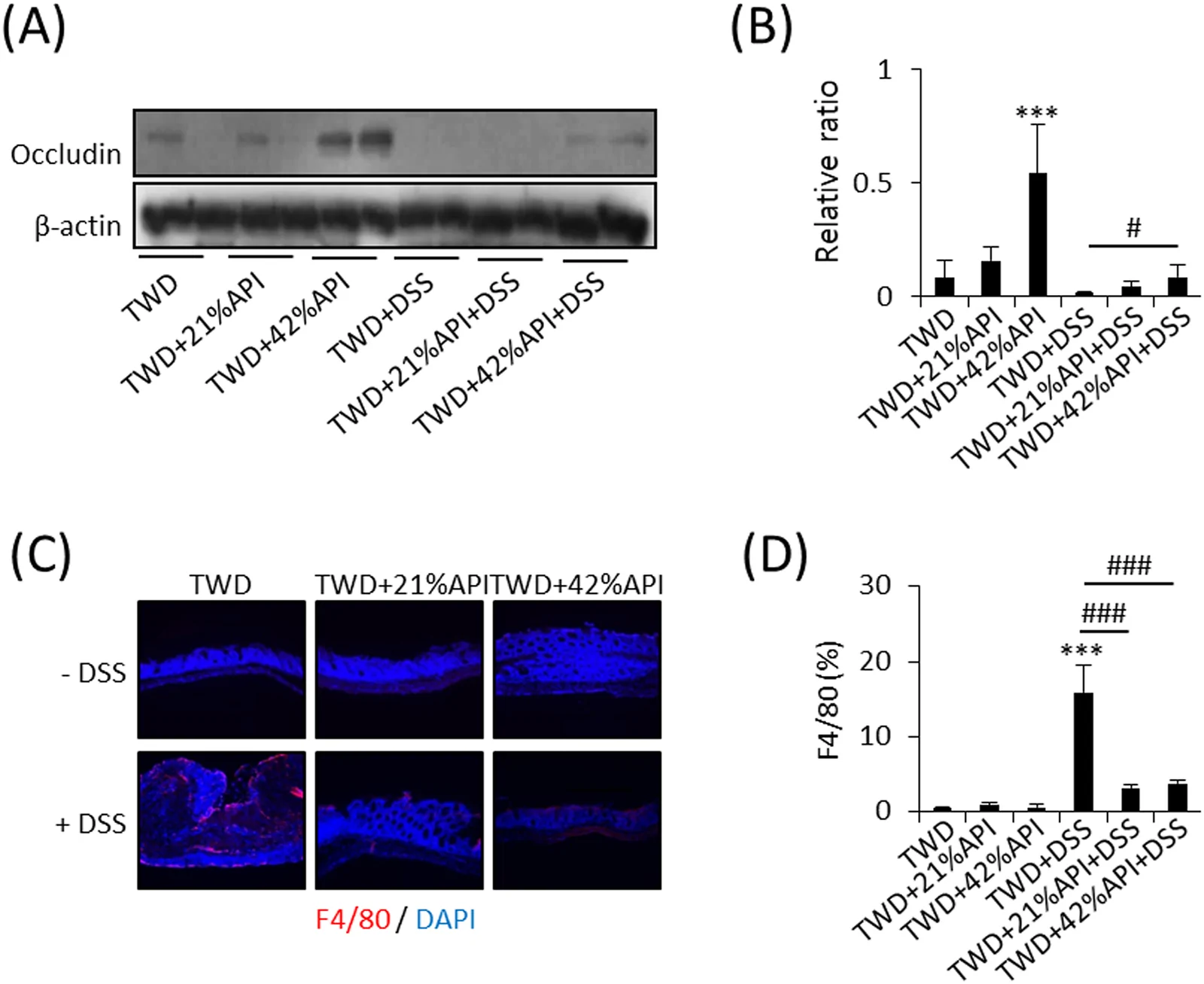
API vegetable supplementation attenuated loss of tight junction protein and immune cell infiltration into the mucosa area induced by DSS treatment. The abundance of colonic occludin was assessed by Western blot (A), and its relative quantity (B) was analyzed by ImageJ software. F4/80-positive cells were detected by immunofluorescence microscopy (C), and the F4/80-positive area was quantified by ImageJ software (D). Data are presented as mean ± SD (*n* = 4 for B; *n* = 3 for C). ∗∗∗*P* < 0.001 compared with TWD. #*P* < 0.05, ###*P* < 0.001 compared with TWD + DSS. API, apiaceous; DAPI, 4′,6-diamidino-2-phenylindole; DSS, dextran sulfate sodium; TWD, total Western diet.

Structural damage was observed in TWD + DSS, which was improved by API supplementation, resulting in an appearance similar to DSS-unchallenged groups (Figure 3A and B**)**. The mucus area stained with Alcian blue (blue area) was barely detectable in TWD + DSS compared to TWD + 21% API + DSS and TWD + 42% API + DSS (Figure 3C and D), indicating that API intake protected the colonic mucus layer. Occludin, a tight junction protein, was barely detected in TWD + DSS but was improved in both TWD + 42% API and TWD + 42% API + DSS (Supplemental Figure 3, Figure 4A and B). Increased F4/80-positive cells were observed in TWD + DSS; however, this was improved by API supplementation (Figure 4C and D). Altogether, API supplementation prevented DSS-induced gut barrier damage and suppressed inflammatory responses, evidenced by suppressed infiltration of F4/80-positive immune cells into colonic mucosa.

### API attenuated TWD/DSS-induced alteration in gut microbial diversity

**FIGURE 5:**
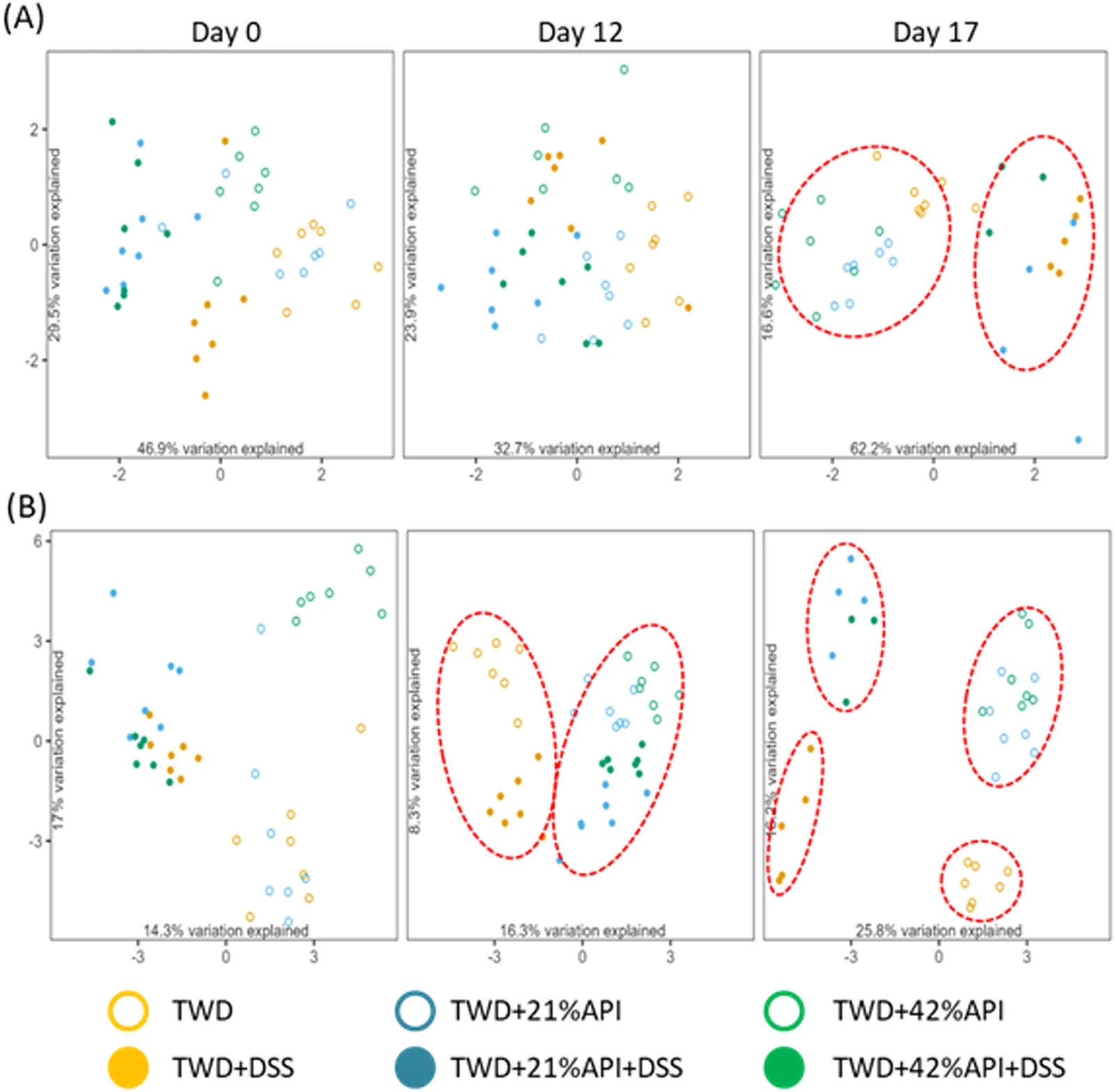
The influence of API supplementation to TWD on gut microbial composition over time before and after DSS treatment. sPLS-DA plots were created at phylum level (A) and genus level (B). Open circle, no DSS treatment group; filled circle, DSS treatment group; yellow circle, TWD; blue circle, TWD + 21% API; green circle, TWD + 42% API. API, apiaceous; DSS, dextran sulfate sodium; sPLS-DA, sparse partial least squares discriminant analysis; TWD, total Western diet.

On day 17, DSS induced clear phylum-level separation in microbial community structure (62.2% variation), whereas API supplementation had no phylum-level effect under DSS challenge (Figure 5A). At the genus level, group separation in bacterial composition increased over time (Figure 5B). In DSS-untreated mice, API supplementation produced distinct clustering from TWD on days 12 and 17; in DSS-challenged mice, API supplementation also separated from TWD + DSS on day 17. Thus, API supplementation altered community structure in both DSS-challenged and unchallenged groups, but 42% API did not differ from 21% API in either condition.

After adjusting day 17 α diversity indices for baseline (day 0), both TWD + 21% API and TWD + 42% API increased ACE, Chao1, Fisher, and observed amplicon sequence variants (ASVs) compared to TWD. Shannon and Simpson metrics increased only in TWD + 21% API (Supplemental Table 8, adjusted *P* value <0.05). Under DSS challenge, both API doses increased Chao1, Shannon, and Simpson metrics compared to TWD + DSS (Supplemental Table 9). No differences in α diversity were observed between 21% and 42% API with or without DSS.

### API feeding promoted beneficial intestinal microbial composition

Bacteroidota and Pseudomonadota (Proteobacteria) were higher in all DSS-challenged groups relative to their respective non-DSS counterparts (Supplemental Figure 4). API supplementation did not affect phylum-level DSS effects. At the genus level, DSS enriched *Bacteroides* and *Parasutterella* but depleted *Lachnospiraceae A2*, *Eubacterium xylanophilum* group, and *Roseburia* across all groups (Supplemental Figure 5, Supplemental Table 10). Comparing TWD + DSS to TWD, DSS increased *Paraclostridium* and *Enterococcus* but decreased *Lachnospiraceae NK4A136 group*, *Muribaculaceae*, and *Turicibacter* (Supplemental Table 10).

In DSS-unchallenged mice, both API doses increased the *Eubacterium xylanophilum* group, *Lachnospiraceae FCS020* group, and *Ruminococcus* compared to TWD, whereas TWD had higher *Bacteroides*, *Dorea*, *Eubacterium fissicatena* group, *Muribaculaceae*, and *Romboutsia* (Supplemental Table 11).

**FIGURE 6:**
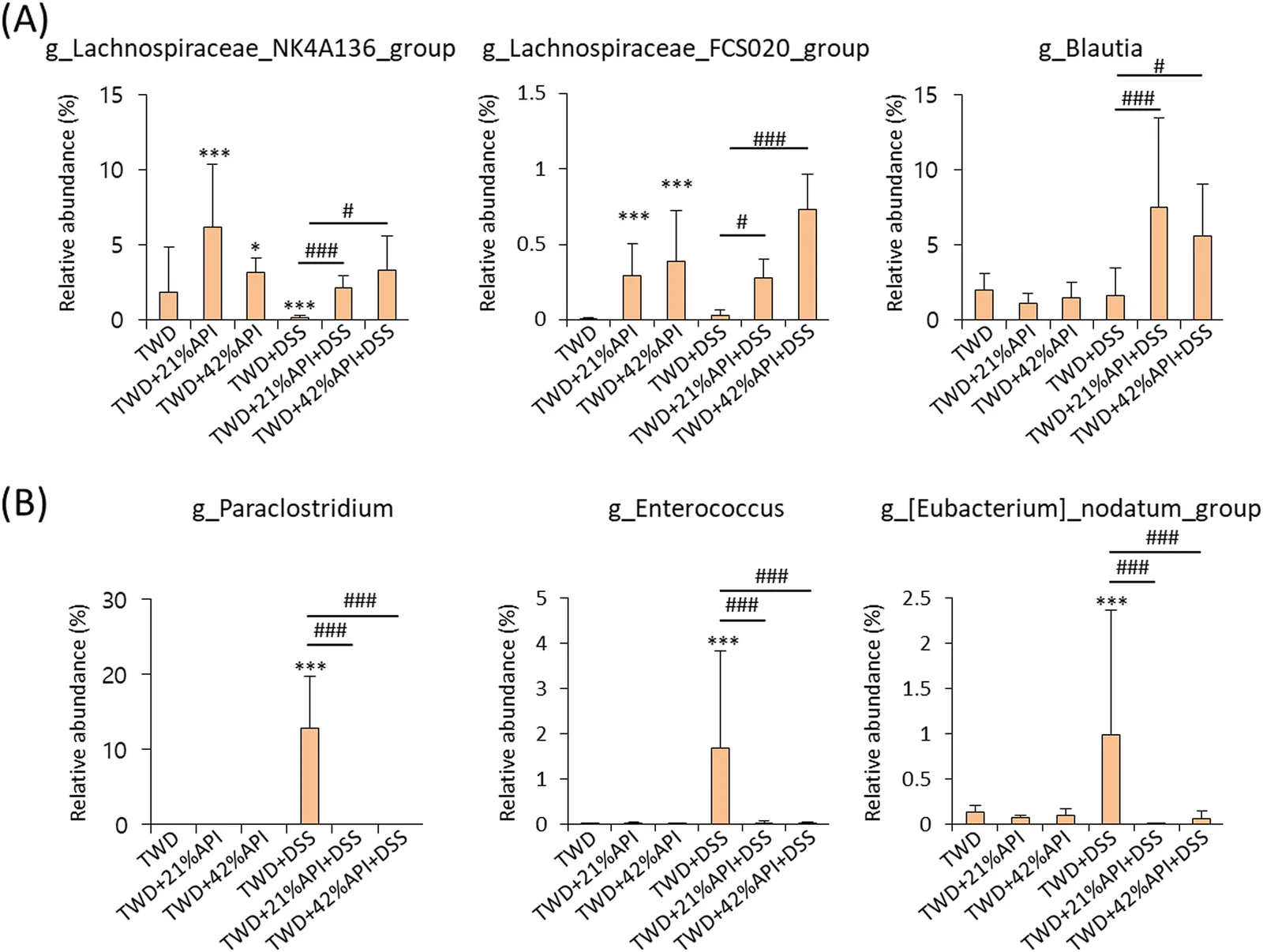
Altered gut bacterial abundance by API supplementation in mice fed TWD and with DSS treatment. (A) Bacteria enriched by API supplementation in DSS-treated mice. (B) Bacteria enriched by DSS treatment but reversed by API supplementation. Data are presented as mean ± SD (*n* = 3‒7). ∗*P* < 0.05, ∗∗∗*P* < 0.001 compared with TWD. #*P* < 0.05, ###*P* < 0.001 compared with TWD + DSS. API, apiaceous; DSS, dextran sulfate sodium; TWD, Total Western Diet.

With the DSS challenge, both API doses increased the abundance of *Blautia*, *Lachnospiraceae FCS020*, *Lachnospiraceae NK4A136*, *Muribaculaceae*, *Oscillibacter*, and *Turicibacter*, and reduced *Adlercreutzia*, *Enterococcus*, *Eubacterium nodatum* group, *Lactobacillus*, and *Paraclostridium* compared to TWD + DSS (Supplemental Table 12). The API-associated enrichment of *Lachnospiraceae NK4A136 group*, *Lachnospiraceae FCS020 group*, and *Blautia* (Figure 6A), along with suppression of DSS-induced *Paraclostridium*, *Enterococcus*, and *Eubacterium nodatum* group (Figure 6B), suggests these taxa contribute to API-mediated protection against DSS-induced colitis.

### Differences in fecal metabolome induced by API intake

**FIGURE 7:**
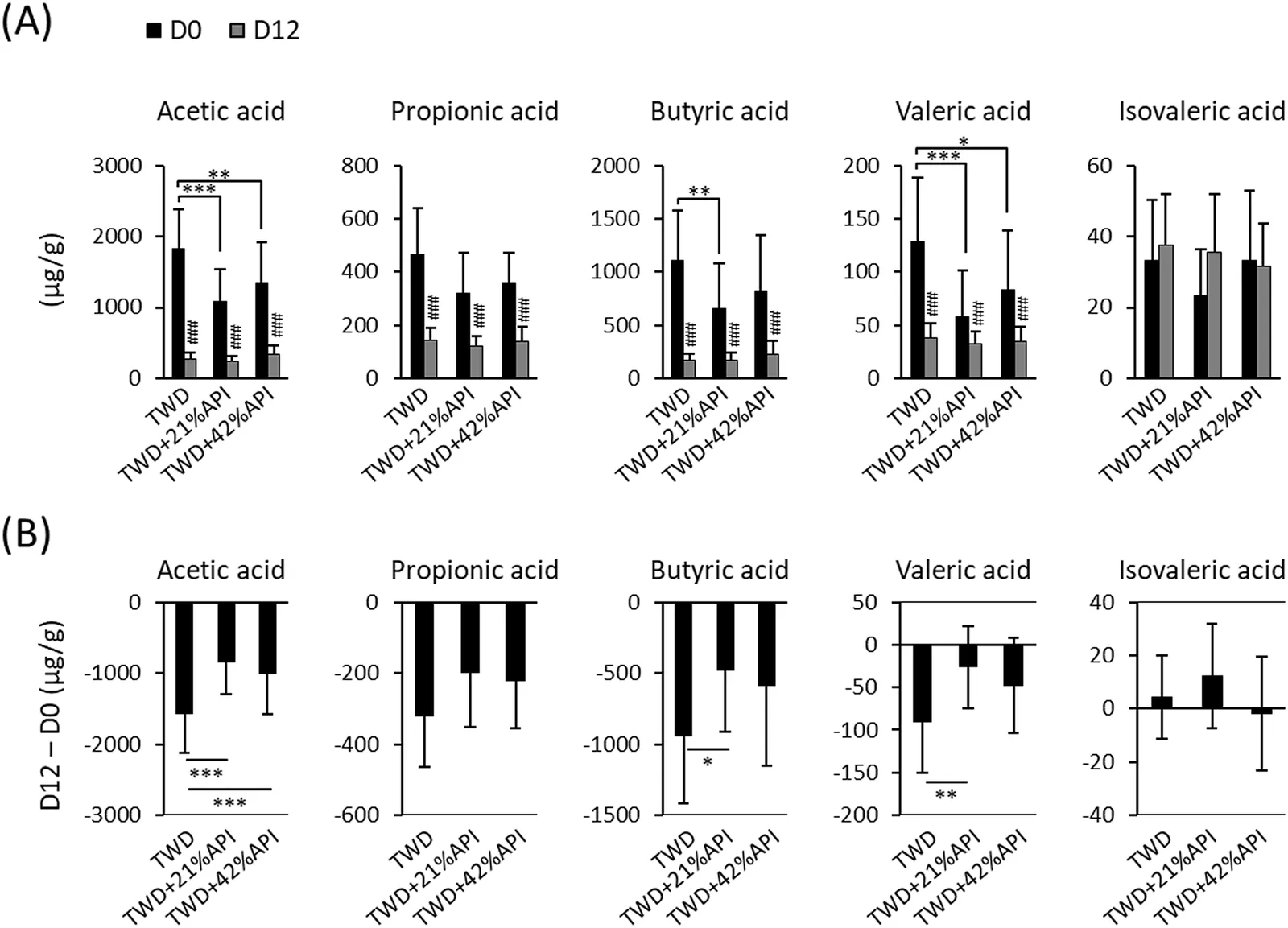
Effect of API intake on short-chain fatty acids during TWD consumption. Fecal samples collected from day 0 (D0) and day 12 (D12) were used for short-chain fatty acid (acetic acid, propionic acid, butyric acid, valeric acid, isovaleric acid) measurement. (A) Individual short-chain fatty acid concentrations at D0 and D12. (B) Change in individual short-chain fatty acid concentrations between D0 and D12. Data are presented as mean ± SD (*n* = 15). ∗*P* < 0.05, ∗∗*P* < 0.01, ∗∗∗*P* < 0.001 compared with TWD (D0). ###*P* < 0.001 between D0 and D12 in each diet groups. API, apiaceous; TWD, total Western diet.

All SCFAs, except for isovaleric acid, were decreased after 12 d of TWD consumption regardless of API intake level (Figure 7A). Although API intake did not increase final SCFA concentrations, when comparing the change in each SCFA from day 0 to day 12 across groups, TWD + 21% API and TWD + 42% API mitigated the reductions in acetic acid compared to TWD-only fed mice. Similarly, TWD + 21% API diminished the reduction in butyric acid and valeric acid compared to TWD-only fed mice (Figure 7B). Altogether, this indicates that API intake ameliorated the reduction in SCFA elicited by TWD intake.

**FIGURE 8:**
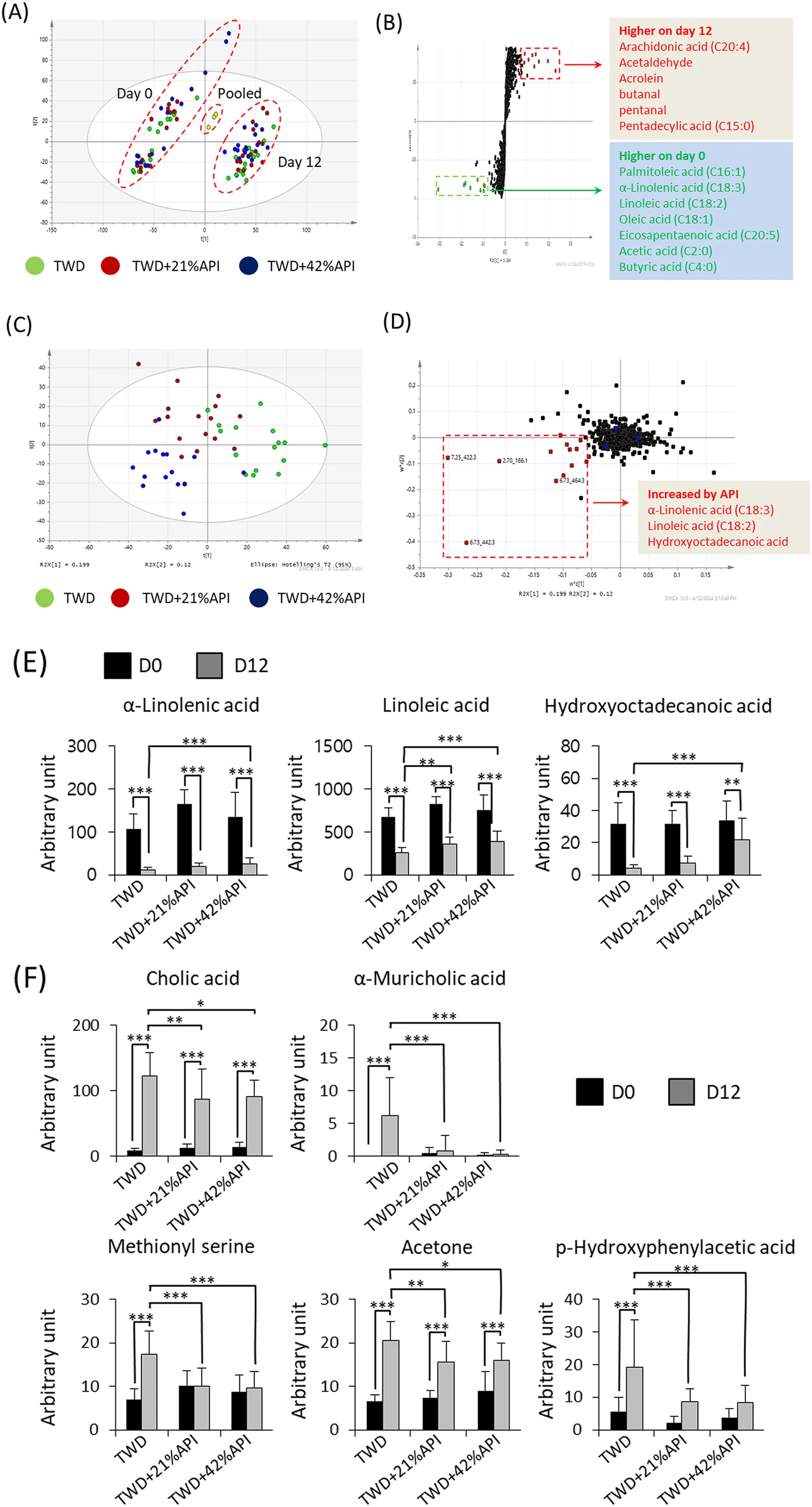
Untargeted metabolomics analysis in feces from day 0 (D0) and day 12 (D12). (A) PCA plot showing differences between D0 and D12. (B) S-plot showing increased and decreased metabolites between D0 and D12 in all 3 diet groups. (C) PLS-DA plot was created to determine the difference in metabolites between groups on D12. (D and E) Identified metabolites that were increased by API supplementation on D12. (F) Identified metabolites that were increased by TWD consumption but reversed by API supplementation. Data are presented as mean ± SD (*n* = 15). API, apiaceous; PCA, principal component analysis; PLS-DA, partial least squares discriminant analysis; TWD, total Western diet.

To elucidate other metabolite changes between day 0 and day 12, untargeted metabolomics analyses were performed. Clear separation in metabolomes was observed between day 0 and day 12 using principal component analysis modeling (Figure 8A). Metabolites contributing to this separation after 12 d of TWD consumption in all diet groups were visualized using an S-plot (Figure 8B and Supplemental Table 13). Arachidonic acid and aldehydes (acetaldehyde, butanal, pentanal, acrolein) were increased, but unsaturated fatty acids (palmitoleic acid, α-linolenic acid, linoleic acid, oleic acid, EPA) and SCFA (acetic acid and butyric acid) were decreased in all 3 groups on day 12, compared to day 0 (Figure 8B and Supplemental Table 13).

Next, to determine the differences in fecal metabolites related to API supplementation in day 12 samples, a PLS-DA model was applied. Fecal metabolites were differentially clustered by each of the TWD, TWD + 21% API, and TWD + 42% API diet groups (Figure 8C). Compared to TWD, α-linolenic acid, linoleic acid, and hydroxyoctadecanoic acid were the enriched metabolites in TWD + 42% API on day 12 (Figure 8D and E). Five metabolites, tentatively identified as cholic acid, α-muricholic acid, methionyl serine, acetone, and p-hydroxyphenylacetic acid based on their accurate mass and elemental composition, were increased by TWD but reversed by API supplementation (Figure 8F).

## Discussion

Diet is a key environmental factor that influences colonic health and gut microbial composition; it is well-known that Western dietary patterns are linked to inflammation-associated diseases and colon cancer [34,35]. TWD was designed to mimic the United States dietary pattern in rodents, as previously well-described [13]. Briefly, TWD contains less complex carbohydrates (but ∼2.6 times more simple sugar) and protein, but approximately twice the fat compared to the typically used American Institute of Nutrition (AIN)-93G rodent diet based on energy density [13]. Also, TWD contains varied sources of fat (soybean oil, milkfat, olive oil, lard, beef tallow, corn oil) compared to only soybean oil in AIN-93G, and is ∼2‒3 times lower in calcium, copper, folate, thiamin, choline, and vitamins B6, B12, D, and E but ∼6 times higher in sodium [13]. TWD can exacerbate DSS-induced colitis and inflammation-associated colorectal cancer development in mice through increased expression of inflammatory genes in colonic mucosa [14,15]. Here, we used TWD as the background diet to mimic the typical United States dietary pattern and found that API supplementation attenuated DSS-induced colitis phenotypes. This suggests that modest API consumption (equivalent to ∼128 g/d for humans) may be beneficial in attenuating colitis.

IL-4 and IL-13 are involved in anti-inflammatory responses, wound healing, and tissue regeneration [36]. Reduced IL-4 and IL-13 levels were observed in inflamed mucosa of UC patients compared to non-inflamed tissue and healthy controls, respectively [37,38]. IL-27 also exerts protective effects against colitis and colitis-associated cancer [39,40], in part by downregulating cytokines (CXCL1, granulocyte-macrophage colony-stimulating factor [GM-CSF], IL-6, TNF-α) [41]. In this study, reduced IL-27 may have contributed to elevated CXCL1 in TWD + DSS. Given that CXCL1 and CXCL9 are associated with UC and colorectal cancer [[42], [43], [44], [45]], the normalization of IL-4, IL-13, and IL-27, and reduced CXCL1 and CXCL9 by API supplementation, may have contributed to colonic protection. This effect may stem from phytochemicals in *Apiaceae,* given that celery and parsnip used here could not appreciably offset the specific micronutrient levels low in TWD. Analytical studies show celery and parsnip contain multiple classes of phytochemicals that could plausibly mediate protective effects of whole-food interventions. These include flavonoids (apigenin, luteolin), phenolic acids (caffeic, p-coumaric), polyacetylenes (falcarinol, falcarindiol), and furanocoumarins (psoralen, bergapten, xanthotoxin) (e.g., [[46], [47], [48], [49]]). Many of these compounds demonstrate biological activity in animal and in vitro models, including anti-inflammatory, cytoprotective, and antioxidant effects, suggesting biologically plausible pathways by which celery and parsnip may confer protection. For example, falcarinol increased plasma IL-2, IL-4, and IL-13 and reduced inflammation in LPS-treated mice [50], whereas apigenin increased tight junction proteins and reduced injury in mice with DSS-induced UC [51]. In LPS-treated RAW264.7 cells, bergapten suppressed TNFα, IL-1β, IL-6, and prostaglandin E2 (PGE2) [52], and xanthotoxin inhibited production of inflammatory cytokines by suppressing activaor protein 1 (AP-1), nuclear factor kappa B (NF-κB), and Janus kinase-signal transducer and activator of transcription (JAK-STAT) signaling [53]. Aged parsnip extract had increased falcarindiol content and decreased acrolein-induced inflammatory cytokines (GM-CSF, IL-23, IL-6, intercellular adhesion molecule 1 [ICAM-1], IL-17, interferon-γ) in mice and in vitro studies [54]. These data suggest that API’s protective effects in DSS colitis are plausibly mediated by anti-inflammatory phytochemicals from API vegetables. Although we did not analyze specific phytochemicals, their profiles are well documented, and our whole-food design improves translational relevance by capturing matrix and synergy effects seen in human diets.

Decreased gut microbial diversity is observed in inflammatory conditions such as UC and CD [55,56] and in DSS-treated mouse models[57,58], consistent with our findings. API supplementation altered microbial community structure and mitigated DSS-induced loss of diversity, suggesting protection against gut dysbiosis. API most abundantly enriched *Lachnospiraceae NK4A136 group* and *Blautia*, while markedly suppressing *Paraclostridium* (the most abundant genus induced by DSS treatment). In our previous study, supplementation of 21% API to both high-fat-diet (HFD) and TWD increased *Lachnospiraceae* in mice [59]. *Blautia* spp. intervention can reduce colonic inflammatory response and oxidative stress, increase cecal SCFA production, improve gut barrier integrity, and remodel microbial community structure in animal models of colitis [60,61]. In the current study, API increased *Blautia* levels despite DSS challenge, implying a prebiotic effect that promotes *Blautia* growth and enrichment, as *Blautia* is known to use fermentable fibers [62,63]. *Lachnospiraceae NK4A136,* a known butyrate-producing bacterium [64], is decreased in various animal models of colitis and CD [[65], [66], [67]]. *Paraclostridium benzoelyticum* is a potent mucin-degrading bacterium [68], and *Paraclostridium bifermentans* exacerbated DSS-induced colitis in mice, evidenced by increased disease activity, deterioration in colon histology, induced colonic inflammatory gene expression (TNFα, IL-1, IL-17), and depleted SCFA in stool [69]. Thus, API-mediated decreases in *Paraclostridium* species and increases in *Lachnospiraceae* and *Blautia* seen here suggest that API’s protective effects against colitis include microbiota modulation.

Moreover, fermented celery juice can increase *Blautia* abundance in mice fed a HFD [70]. Interestingly, whole celery and its soluble dietary fiber (primarily pectin), flavonoids, and insoluble dietary fiber (primarily cellulose) all attenuated DSS-induced colitis phenotypes in mice (colon shortening, disease activity, serum IL-1β, intestinal barrier function) and enriched *Blautia* species [71]. The soluble fiber also suppressed the DSS-induced growth of *Paraclostridium* [71]. These findings imply that soluble fiber in celery may contribute to the protective role of API against DSS-induced colitis in mice fed TWD through promoting growth of *Blautia* and suppressing *Paraclostridium* species. Additionally, diet supplementation with falcarinol and falcarindiol in a rat model of colorectal cancer also showed modification of gut microbiota composition [72]. Apigenin administration to UC-induced mice also resulted in recovery of α diversity, reversal of DSS-induced changes in abundance of various genera, and improvement in colonic SCFA content [51]. Collectively, the data suggest that the specific composition of API vegetables, including phytochemicals, may be of unique benefit to gut-related health. Nonetheless, the comparisons in the current study were limited to API compared with no-vegetable controls, without inclusion of non-API vegetables.

Arachidonic acid is a main source of pro-inflammatory prostaglandins and leukotrienes, which are increased in IBD patients [73]. High intake of arachidonic acid (240 mg/kg) exacerbated DSS-induced colitis in rats [74]. In the current study, TWD consumption increased fecal arachidonic acid by day 12 (Supplemental Table 13), likely reflecting the high content of animal-derived fats in this diet. Acrolein is a toxic aliphatic aldehyde that exists in various foods (e.g., French fries, potato chips) and can be generated from triglycerides (via hydrolysis and free radical pathways) and from polyunsaturated fatty acids (e.g., arachidonic acid, linolenic acid, and linoleic acid) via lipid peroxidation pathways [75]. The concomitant increase in fecal aldehydes (e.g., acrolein, acetaldehyde, pentanal) and the significant depletion of dietary PUFAs, such as α-linolenic acid (C18:3) and EPA (C20:5) (Supplemental Table 13), suggests that the TWD increases oxidative stress in the gut. This pattern suggests peroxidation of the collective fecal PUFA pool, in which the dietary influx of arachidonic acid likely serves as a significant substrate. These findings align with previous reports that HFD consumption increases fecal pro-inflammatory metabolites, including acetone, which have been shown to directly trigger inflammatory cytokine production in vitro [76]. The TWD-induced increase in acetone observed here likely contributes to the colitis phenotype by systemically triggering low-grade chronic inflammation, an effect that was attenuated by API supplementation.

Moreover, *Coriobacteriaceae* *uncultured group (UCG)-002* and *Turicibacter* were the most abundantly enriched bacteria in all 3 diet groups on day 12 (Supplemental Figure 6). Supporting this, *Coriobacteriaceae* was enriched by HFD consumption in mice and promoted colorectal tumorigenesis [77]. Similarly, others reported *Turicibacter spp.* were enriched in mice with chronic ethanol consumption [78]. Although *Turicibacter* is often associated with leanness and improved metabolic health [79], its enrichment in response to TWD here is notable. This may be explained by strain-specific effects; for instance, although some *Turicibacter* strains are linked to anti-obesity effects [79], others have been shown to promote adiposity in mice [80]. Given that our 16S rRNA sequencing does not provide strain-level resolution, the observed increase may reflect the expansion of TWD-responsive strains.

Untargeted metabolomics in our study suggested that API reversed TWD-induced increases in bile acid metabolites, including cholic acids, which is potentially driven by bile acid deconjugation mediated by Turicibacter bile salt hydrolase [80]. Mono-colonization of *Turicibacter* strains in germ-free mice increased cecal cholic acid and β-muricholic acid [80]. Although our metabolomics analyses were conducted on fecal samples, the enrichment of these same bile acids in TWD-fed mice suggests a similar metabolic shift within the intestinal tract that may be related to our observed enrichment of *Turicibacter*. Similairly, 2 large prospective cohort studies observed an association between serum concentrations of downstream microbial metabolites of cholic acid and risk of colorectal cancer, particularly in females [81]. Further, we speculate that the attenuation of increased bile acids in the feces by API supplementation suggests that API may influence the enzymatic activity or metabolic niche of *Turicibacter* related to bile acid metabolism; however, further follow-up studies are needed to confirm this observation.

In summary, supplementation of celery and parsnip to TWD attenuated DSS-induced colitis, decreased inflammatory markers, and favorably modulated the gut microbiota and metabolome. We acknowledge that the housing density of 7–8 mice per cage is a limitation of this study, as cohousing can lead to cage-specific microbiota shifts due to coprophagy and the shared environment, which likely contributed to the observed baseline variation in SCFAs (day 0). Although the experimental unit in such designs is often considered the cage rather than the individual mouse, the consistency of the protective trends observed across multiple primary endpoints—including histological scores and inflammatory markers—suggests that the effects of API supplementation are robust. Furthermore, although the limited fecal volume precluded metabolomic analyses at day 17, the significant metabolic shifts observed at day 12 provide insight into the pre-DSS state. The API supplementation suppressed DSS-induced pathogenic bacteria (mainly *Paraclostridium*) and promoted beneficial bacteria (mainly *Lachnospiraceae* and *Blautia*). TWD consumption increased fecal arachidonic acid and aliphatic aldehydes before DSS challenge, suggesting these TWD-derived fecal metabolites may contribute to the TWD-exacerbated colitis and colorectal cancer risk previously reported [15]. API did not counteract these specific changes but did attenuate the TWD-induced reductions in some SCFA. API supplementation also lessened TWD-induced increases in cholic acid, α-muricholic acid, methionyl serine, acetone, and *p*-hydroxyphenylacetic acid, some of which may increase risk of colon inflammation and cancer. These protective effects may be attributable to the fiber and phytochemical composition of API vegetables achieved at intake levels equivalent to ∼128 g/d for humans. However, further investigation (including direct comparisons with non-API vegetables) is warranted to fully elucidate mechanisms by which API protects against colon inflammation and, by extension, colon cancer risk as well as other chronic diseases associated with a Western-style dietary pattern.

## Author contributions

The authors’ responsibilities were as follows – H-SL, SPT: designed research; H-SL, LEI, BZ, CC: conducted research; H-SL, JZ, RF, QDR, MG, LY, and CC: analyzed data or performed statistical analysis; H-SL: wrote article; SPT: had primary responsibility for final content; and all authors: read and approved the final manuscript.

## Data availability

Data described in the manuscript will be made available upon request to the corresponding author.

## Declaration of generative AI and AI-Assisted technologies in the writing process

During the preparation of this work, the author(s) used Gemini (Google) in order to assist with improving conciseness and readability of portions of the manuscript during revision. After using this tool, the authors reviewed and edited the content as needed and take full responsibility for the content of the publication.

## Funding

SPT, H-SL, and LEI were supported by the 21st Century Endowed Chair in Human Environmental Sciences, University of Arkansas-Fayetteville. LY and MG were supported by USDA- Agricultural Research Service (6026-51000-012-000D and 6026-10700-001-00D). JZ was supported by the USDA National Institute of Food and Agriculture, Agriculture and Food Research Initiative Competitive grant number 20196701629869, and funds from the Arkansas Biosciences Institute. CC was supported by the Minnesota Agricultural Experiment Station (MIN-18-150). Supporting sources had no involvement in study design; collection, analysis, and interpretation of data; writing of the report; or any restrictions regarding the submission of the report for publication.

## Conflict of interest

The authors report no conflicts of interest.
