# Supplementary material for: Protective Effect of Apiaceous Vegetables against Total Western Diet- and Dextran Sulfate Sodium-Induced Colitis in Mice
Source: J Nutr. 2026 Jun 7;156(8):101652. doi: 10.1016/j.tjnut.2026.101652 (PMC13494232; doi:10.1016/j.tjnut.2026.101652)
Supplement: Supplementary file 1 [file mmc1.docx]

**Supplementary Material**

Protective effect of apiaceous vegetables against Total Western Diet- and dextran sulfate sodium-induced colitis in mice

First Author: Hee-Seop Lee^1^

Corresponding Author: Sabrina P. Trudo^1^*

^1^ School of Human Environmental Sciences, University of Arkansas, Fayetteville, AR, 72701, USA

*E-mail address: trudo@uark.edu

Supplementary Figure 1. Information of each spot on the membranes of cytokine and chemokine array. Red-colored features indicate the selected cytokines and chemokines for heatmap. Abbreviations: C5a, complement component 5a; CC, chemokine C-C motif ligand; CCL, chemokine C-C motif ligand; CXCL and CXL, C-X-C motif ligand; GM-CSF, granulocyte-macrophage colony-stimulating factor; ICAM-1, intercellular adhesion molecule 1; IFN, interferon; IL, interleukin; TIMP, tissue inhibitor of metalloproteinase; TNF, tumor necrosis factor; TREM, triggering receptor expressed on myeloid cells.

Supplementary Figure 2. Original blot images of cytokine array. Blots A in red box were presented in Figure 2C. Abbreviations: API, apiaceous vegetables; DSS, dextran sulfate sodium; TWD, Total Western Diet.

Supplementary Figure 3. Original blot images of occludin.

Supplementary Figure 4. Bacterial composition changes over time at phylum level in DSS-induced colitis mice fed TWD supplemented with or without API. Fecal samples were collected from Day 0 (baseline, before TWD feeding started), Day 12 (before DSS treatment), and Day 17 (after DSS treatment) for 16s rRNA sequencing. G1, TWD; G2, TWD+21%API; G3, TWD+42%API; G4, TWD+DSS; G5, TWD+21%API+DSS; G6, TWD+42%API+DSS. Abbreviations: API, apiaceous vegetables; DSS, dextran sulfate sodium; TWD, Total Western Diet.

Supplementary Figure 5. Bacterial composition changes over time at genus level in DSS-induced colitis mice fed TWD supplemented with or without API. Fecal samples were collected from Day 0 (baseline, before TWD feeding started), Day 12 (before DSS treatment), and Day 17 (after DSS treatment) for 16s rRNA sequencing. Top 30 genera are presented. G1, TWD; G2, TWD+21%API; G3, TWD+42%API; G4, TWD+DSS; G5, TWD+21%API+DSS; G6, TWD+42%API+DSS. Abbreviations: API, apiaceous vegetables; DSS, dextran sulfate sodium; TWD, Total Western Diet.

Supplementary Figure 6. The most abundantly enriched bacteria after consumption of TWD with or without API for 12 days. Data are presented as mean ± S.D. (n=15). ***p<0.001 (D0 vs D12). Abbreviations: API, apiaceous vegetables; TWD, Total Western Diet.

Supplementary Table 1. Diet composition of Teklad rodent diet (#8604, Inotiv Inc.) during acclimation period.

| Macronutrients |  |  | Vitamins |  |
| --- | --- | --- | --- | --- |
| Carbohydrates (%) | 40.2 |  | Vitamin A (IU/g) | 12.6 |
| Protein (%) | 24.3 |  | Vitamin D3 (IU/g) | 2.4 |
| Fat (%) | 4.7 |  | Vitamin E (IU/g) | 120 |
| Fiber (%) | 16.4 |  | Vitamin K3 (menadione) (mg/kg) | 40 |
| Energy density (kcal/g) | 3.0 |  | Vitamin B1 (thiamin) (mg/kg) | 27 |
| Calories from carbohydrate (%) | 54 |  | Vitamin B2 (riboflavin) (mg/kg) | 8 |
| Calories from protein (%) | 32 |  | Niacin (nicotinic acid) (mg/kg) | 63 |
| Calories from fat (%) | 14 |  | Vitamin B6 (pyridoxine) (mg/kg) | 13 |
|  |  |  | Pantothenic acid (mg/kg) | 21 |
| Minerals |  |  | Vitamin B12 (cyanocobalamin) (mg/kg) | 0.05 |
| Calcium (%) | 1.4 |  | Biotin (mg/kg) | 0.38 |
| Phosphorous (%) | 1.1 |  | Folate (mg/kg) | 3 |
| Sodium (%) | 0.3 |  | Choline (mg/kg) | 2530 |
| Potassium (%) | 1.0 |  |  |  |
| Chloride (%) | 0.5 |  |  |  |
| Magnesium (%) | 0.3 |  |  |  |
| Zinc (mg/kg) | 80 |  |  |  |
| Manganese (mg/kg) | 100 |  |  |  |
| Copper (mg/kg) | 25 |  |  |  |
| Iodine (mg/kg) | 2 |  |  |  |
| Iron (mg/kg) | 300 |  |  |  |
| Selenium (mg/kg) | 0.34 |  |  |  |

Information of Teklad rodent diet (#8604) was provided by Inotiv Inc. (Indianapolis, IN). Ingredients: Dehulled soybean meal, wheat middlings, flaked corn, ground corn, fish meal, cane molasses, ground wheat, dried whey, soybean oil, brewers dried yeast, dicalcium phosphate, calcium carbonate, iodized salt, choline chloride, magnesium oxide, ferrous sulfate, vitamin E acetate, menadione sodium bisulfite complex (source of vitamin K activity), manganous oxide, copper sulfate, zinc oxide, niacin, thiamin mononitrate, vitamin A acetate, vitamin D3 supplement, calcium pantothenate, pyridoxine hydrochloride, riboflavin, vitamin B12 supplement, calcium iodate, folic acid, biotin, cobalt carbonate.

Supplementary Table 2. Diet ingredients of vegetable-free and vegetable-containing TWD.

|  | TWD |  | TWD+21%API | TWD+42%API |
| --- | --- | --- | --- | --- |
| Cornstarch (g) | 230.0 |  | 208.0 | 186.0 |
| Casein (g) | 190.0 |  | 188.0 | 186.0 |
| Dextrinized cornstarch (g) | 70.0 |  | 70.0 | 70.0 |
| Sucrose (g) | 261.4 |  | 261.4 | 261.4 |
| Oil mixture (g)^a^ | 165.4 |  | 164.9 | 164.4 |
| Fiber (cellulose) (g) | 30.0 |  | 23.2 | 16.3 |
| Mineral mix (g)^b^ | 35.0 |  | 35.0 | 35.0 |
| Vitamin mix (g)^c^ | 10.0 |  | 10.0 | 10.0 |
| L-cystine (g) | 2.9 |  | 2.9 | 2.9 |
| Choline bitartrate (g) | 1.4 |  | 1.4 | 1.4 |
| Sodium chloride (g) | 4.0 |  | 4.0 | 4.0 |
| Celery (g)^d^ | 0.0 |  | 105.0 | 210.0 |
| Parsnip (g)^d^ | 0.0 |  | 105.0 | 210.0 |
| Water (g)^e^ | 367.3 |  | 183.6 | 0.0 |
| Total (g) | 1137.3 |  | 1154.3 | 1171.4 |
| % Carbohydrate^d^ | 49.4 |  | 48.6 | 47.9 |
| % Protein^d^ | 16.7 |  | 16.5 | 16.2 |
| % Fat^d^ | 14.5 |  | 14.3 | 14.1 |
| % Dietary fiber^d^ | 2.6 |  | 2.6 | 2.6 |
| Calories (kcal/g diet) | 4.0 |  | 3.9 | 3.8 |

^a^Fat composition of oil mixture is described in Supplementary Table 3. ^b^Mineral composition is described in Supplementary Table 4. ^c^Vitamin composition is described in Supplementary Table 5. ^d^Fresh weight of the vegetable. ^d^Carbohydrate, protein, fat, and dietary fiber from vegetables were calculated as indicated in the United States Department of Agriculture food composition database. For celery, carbohydrate, 2.97 g/100 g; protein, 0.69 g/100 g; fat, 0.17 g/100 g; fiber, 0.75 g/100 g; water, 95.4 g/100 g. For parsnip, carbohydrate, 18 g/100 g; protein, 1.2 g/100 g; fat, 0.3 g/100 g; fiber, 4.9 g/100 g; water, 79.5 g/100 g. ^e^Because of water content of the vegetables, equivalent amount of water to the vegetables was added to the TWD and TWD+21%API diet to match water content across all diets and achieve similar thick paste-like texture of TWD+42%API diet. Abbreviations: API, apiaceous vegetables; TWD, Total Western Diet.

Supplementary Table 3. Fat composition of TWD^a^.

| Fat (g/kg diet) | TWD |
| --- | --- |
| Soybean oil | 31.4 |
| Anhydrous milk fat | 36.3 |
| Olive oil | 28 |
| Lard | 28 |
| Beef tallow | 24.8 |
| Corn oil | 16.5 |
| Cholesterol | 0.4 |
| Total weight (g) | 165.4 |

^a^The oil mixture of TWD was formulated as described by Hintze et al (13). Abbreviations: TWD, Total Western Diet.

Supplementary Table 4. Mineral mix composition of TWD^a^.

| Minerals (mg/kg diet) | TWD |
| --- | --- |
| Calcium | 2,011 |
| Phosphorus | 2,757 |
| Sodium | 7,078 |
| Potassium | 5,333 |
| Magnesium | 589 |
| Iron | 31 |
| Zinc | 24.6 |
| Copper | 2.6 |
| Selenium | 0.2 |
| Chloride | 10,800 |
| Sulfur | 300 |
| Manganese | 10.5 |
| Iodine | 0.2 |
| Molybdenum | 0.15 |
| Silicon | 5 |
| Chromium | 1 |
| Fluoride | 1 |
| Nickel | 0.5 |
| Boron | 0.5 |
| Lithium | 0.1 |
| Vanadium | 0.1 |
| Total weight (g) | 28,945.45 |

^a^The mineral mixture of TWD was formulated as described by Hintze et al (13). Abbreviations: TWD, Total Western Diet.

Supplementary Table 5. Vitamin mix composition of TWD^a^.

| Vitamins (Unit/kg diet) | TWD |
| --- | --- |
| Vitamin B1 (Thiamin) (mg) | 3.5 |
| Vitamin B2 (Riboflavin) (mg) | 4.4 |
| Niacin (nicotinic acid) (mg) | 50.6 |
| Vitamin B6 (Pyridoxine) (mg) | 3.9 |
| Folate (mg) | 1.3 |
| Vitamin B12 (cyanocobalamin) (μg) | 11 |
| Vitamin A (IU) | 4,318 |
| Vitamin D (IU) | 400 |
| Vitamin E (IU) | 25 |
| Vitamin K (μg) | 189 |
| Choline (mg) | 648 |
| Pantothenic acid (mg) | 15 |
| Biotin (mg) | 0.2 |
| Total weight (g) | 5,669.9 |

^a^The vitamin mixture of TWD was formulated as described by Hintze et al (13). Abbreviations: TWD, Total Western Diet.

Supplementary Table 6. Optimal parameters selected for sPLS-DA models by cross-validation.

| Taxonomic level | Time point | Number of components | Number of x-variables (taxa) retained in model for each component |
| --- | --- | --- | --- |
| Phylum | Day 0 | 2 | 4, 2 |
|  | Day 12 | 2 | 2, 3 |
|  | Day 17 | 3 | 5, 5, 5 |
| Genus | Day 0 | 4 | 70, 70, 50, 10 |
|  | Day 12 | 4 | 10, 20, 20, 20, 10 |
|  | Day 17 | 5 | 20, 70, 50, 70, 60 |

Abbreviations: sPLS-DA, sparse partial least squares discriminant analysis.

Supplementary Table 7. Significantly altered cytokines and chemokines by TWD or API supplementation.

|  | TWD | TWD+21%API | TWD+42%API | TWD+DSS | TWD+21%API+DSS | TWD+42%API+DSS |
| --- | --- | --- | --- | --- | --- | --- |
| CXCL13 | 1.0±0.0 | 1.1±0.0 | 0.8±0.1* | 1.9±0.2*** | 1.1±0.1### | 1.1±0.0### |
| C5a | 1.0±0.0 | 0.9±0.1 | 0.8±0.1 | 0.7±0.3 | 1.0±0.1 | 1.1±0.0## |
| G-CSF | 1.0±0.0 | 0.8±0.1 | 0.5±0.2** | 2.2±0.3*** | 1.1±0.1### | 1.2±0.1### |
| GM-CSF | 1.0±0.1 | 0.5±0.0*** | 0.5±0.1*** | 0.5±0.1*** | 0.3±0.0*** | 0.2±0.0***,## |
| CCL1 | 1.0±0.1 | 0.9±0.1 | 0.4±0.1*** | 0.7±0.3 | 0.9±0.0 | 0.7±0.1 |
| CCL11 | 1.0±0.1 | 0.8±0.1 | 0.7±0.1 | 0.7±0.0 | 0.5±0.1 | 0.7±0.5 |
| ICAM-1 | 1.0±0.0 | 1.0±0.0 | 0.9±0.1* | 0.9±0.0*** | 0.9±0.0* | 0.9±0.0* |
| IFN-γ | 1.0±0.0 | 0.8±0.1** | 0.6±0.1*** | 0.5±0.1*** | 0.6±0.0*** | 0.5±0.1*** |
| IL-1α | 1.0±0.1 | 0.9±0.1 | 0.6±0.1*** | 0.5±0.1*** | 0.6±0.1*** | 0.4±0.0*** |
| IL-1β | 1.0±0.1 | 0.7±0.2 | 0.5±0.1*** | 0.4±0.1*** | 0.8±0.1### | 0.4±0.1*** |
| IL-1γa | 1.0±0.2 | 0.9±0.1 | 0.5±0.1*** | 0.9±0.2 | 0.9±0.2 | 0.5±0.1** |
| IL-2 | 1.0±0.2 | 0.9±0.1 | 0.4±0.1*** | 0.2±0.1*** | 0.9±0.2## | 0.7±0.3# |
| IL-3 | 1.0±0.1 | 1.2±0.1 | 1.0±0.1 | 0.6±0.2* | 0.8±0.1 | 1.0±0.2# |
| IL-4 | 1.0±0.1 | 1.0±0.0 | 0.9±0.1 | 0.3±0.2*** | 0.8±0.1### | 1.0±0.1### |
| IL-5 | 1.0±0.3 | 0.6±0.0 | 0.9±0.2 | 0.8±0.2 | 0.4±0.1** | 0.7±0.1 |
| IL-6 | 1.0±0.1 | 0.9±0.1 | 1.1±0.2 | 1.0±0.2 | 0.8±0.2 | 0.9±0.3 |
| IL-7 | 1.0±0.1 | 1.3±0.2 | 0.9±0.2 | 0.5±0.0** | 1.1±0.3## | 1.1±0.1### |
| IL-10 | 1.0±0.1 | 1.1±0.2 | 1.2±0.4 | 0.9±0.1 | 0.9±0.2 | 0.9±0.2 |
| IL-13 | 1.0±0.1 | 1.1±0.2 | 0.9±0.2 | 0.5±0.0** | 0.9±0.2## | 1.1±0.1### |
| IL-12p70 | 1.0±0.3 | 1.0±0.2 | 1.2±0.3 | 0.6±0.2 | 0.8±0.3 | 1.2±0.2# |
| IL-16 | 1.0±0.1 | 1.2±0.1* | 1.2±0.1 | 1.5±0.1*** | 1.3±0.1** | 1.3±0.1** |
| IL-17A | 1.0±0.1 | 1.2±0.1* | 1.0±0.1 | 0.5±0.1*** | 1.0±0.2### | 0.9±0.1## |
| IL-23 | 1.0±0.2 | 1.0±0.0 | 0.8±0.2 | 0.4±0.1*** | 0.9±0.3## | 0.7±0.1 |
| IL-27 | 1.0±0.1 | 0.9±0.1 | 0.7±0.0** | 0.3±0.1*** | 1.0±0.1### | 1.0±0.1### |
| CXCL10 | 1.0±0.1 | 1.2±0.1 | 0.8±0.1 | 1.0±0.2 | 0.7±0.1**,# | 0.7±0.1**,## |
| CXCL11 | 1.0±0.0 | 0.8±0.1 | 0.6±0.1*** | 0.6±0.2*** | 0.7±0.0* | 0.7±0.1** |
| CXCL1 | 1.0±0.1 | 1.1±0.4 | 0.7±0.2 | 3.1±0.3*** | 2.0±0.2*,# | 1.9±1.0**,# |
| M-CSF | 1.0±0.1 | 1.0±0.0 | 0.9±0.0* | 0.7±0.0*** | 0.9±0.1*,### | 0.9±0.0***,# |
| CCL2 | 1.0±0.1 | 1.2±0.1 | 0.9±0.0 | 1.0±0.0 | 0.9±0.1 | 1.0±0.3 |
| CCL12 | 1.0±0.3 | 1.0±0.1 | 0.7±0.2 | 1.3±0.8 | 1.1±0.1 | 1.1±0.2 |
| CXCL9 | 1.0±0.1 | 1.0±0.0 | 0.6±0.1*** | 2.2±0.1*** | 0.6±0.1***,### | 0.6±0.1***,### |
| MIP-1α | 1.0±0.1 | 0.8±0.2 | 0.5±0.1*** | 0.3±0.0*** | 0.6±0.0***,## | 0.5±0.1*** |
| MIP-1β | 1.0±0.2 | 1.0±0.2 | 0.7±0.1 | 0.7±0.1 | 0.8±0.1 | 0.7±0.1* |
| MIP-2 | 1.0±0.1 | 1.0±0.2 | 0.5±0.0*** | 1.0±0.1 | 1.0±0.1 | 0.7±0.2* |
| CCL5 | 1.0±0.1 | 0.8±0.2 | 0.6±0.0*** | 0.4±0.1*** | 0.8±0.1### | 0.5±0.0*** |
| CXCL12 | 1.0±0.0 | 0.9±0.0** | 0.6±0.1*** | 0.6±0.0*** | 0.9±0.0### | 0.9±0.0**,### |
| CCL17 | 1.0±0.1 | 1.1±0.4 | 0.6±0.0* | 0.4±0.2** | 0.4±0.1** | 0.4±0.1** |
| TIMP-1 | 1.0±0.0 | 1.1±0.1 | 0.9±0.1 | 1.1±0.1 | 1.0±0.0 | 1.0±0.0 |
| TNF-α | 1.0±0.0 | 0.8±0.1* | 0.5±0.1*** | 0.5±0.1*** | 0.6±0.0*** | 0.6±0.1*** |
| TREM-1 | 1.0±0.1 | 0.8±0.1 | 0.6±0.1*** | 0.6±0.1*** | 0.7±0.0** | 0.5±0.1*** |

Each spot in the blot images (Fig. 2C) was quantified by Image J software and the relative cytokine/chemokine levels were analyzed. Relative expression level of the selected chemokines and cytokines, compared to TWD group, are presented as mean ± S.D. *p<0.05, **p<0.01, ***p<0.001 versus TWD. #p<0.05, ##p<0.01, ###p<0.001 versus TWD+DSS. Abbreviations: API, apiaceous vegetables; C5a, complement component 5a; CCL, chemokine C-C motif ligand; CXCL, C-X-C motif ligand; DSS, dextran sulfate sodium; GM-CSF, granulocyte-macrophage colony-stimulating factor; ICAM-1, intercellular adhesion molecule 1; IFN, interferon; IL, interleukin; MIP, macrophage inflammatory protein; TIMP, tissue inhibitor of metalloproteinase; TNF, tumor necrosis factor; TREM, triggering receptor expressed on myeloid cells; TWD, Total Western Diet.

Supplementary Table 8. α-Diversity of mice fed TWD or TWD and supplemented with 21% or 42% API. Diversity was calculated at D17 from stool samples. Data were adjusted for repeated measures and baseline (D0) diversity metrics, and group differences were determined using the Tukey multiple comparison test with the Sidak adjustment. Data are presented as means (95% CI) and the means without a common letter in the row differ significantly.

| α-Diversity index | TWD | TWD+21%API | TWD+42%API |
| --- | --- | --- | --- |
| ACE | 130.1 (122, 138.1) ^a^ | 154.5 (146.5, 162.6) ^b^ | 152.1 (144, 160.1) ^b^ |
| Chao1 | 119.8 (112.7, 127) ^a^ | 140.5 (133.4, 147.7) ^b^ | 139.9 (132.7, 147) ^b^ |
| Fisher | 21.54 (19.94, 23.15) ^a^ | 26.06 (24.46, 27.66) ^b^ | 25.77 (24.17, 27.37) ^b^ |
| Observed | 130.7 (122.5, 138.9) ^a^ | 155.4 (147.2, 163.5) ^b^ | 151.4 (143.2, 159.6) ^b^ |
| Shannon | 2.785 (2.627, 2.943) ^a^ | 3.011 (2.852, 3.169) ^b^ | 2.983 (2.825, 3.141) ^ab^ |
| Simpson | 0.8346 (0.8098, 0.8593) ^a^ | 0.8702 (0.8455, 0.895) ^b^ | 0.8632 (0.8385, 0.8879) ^ab^ |

Abbreviations: ACE, Abundance-based Coverage Estimator; API, apiaceous vegetables; TWD, Total Western Diet.

Supplementary Table 9. α-diversity of mice fed TWD and challenged with DSS or fed TWD supplemented with 21% or 42% API and challenged with DSS. Diversity was calculated at D17 from stool samples. Data were adjusted for repeated measures and baseline (D0) diversity metrics, and group differences were determined using the Tukey multiple comparison test with the Sidak adjustment. Data are presented as means (95% CI) and the means without a common letter in the row differ significantly.

| α-Diversity index | TWD+DSS | TWD+21%API+DSS | TWD+42%API+DSS |
| --- | --- | --- | --- |
| ACE | 126.9 (118.4, 135.4)^a^ | 134.3 (125.4, 143.2)^a^ | 134.1 (124.5, 143.6)^a^ |
| Chao1 | 114.3 (106.8, 121.9)^a^ | 126.1 (118.2, 134)^b^ | 126.9 (118.5, 135.4)^b^ |
| Fisher | 20.68 (18.99, 22.38)^a^ | 22.83 (21.06, 24.6)^a^ | 23.12 (21.22, 25.02)^a^ |
| Observed | 126.2 (117.5, 134.8)^a^ | 132.6 (123.6, 141.7)^a^ | 133 (123.3, 142.7)^a^ |
| Shannon | 2.799 (2.626, 2.973)^a^ | 3.104 (2.93, 3.278)^b^ | 3.185 (3, 3.37)^b^ |
| Simpson | 0.8507 (0.8249, 0.8766)^a^ | 0.8884 (0.8616, 0.9152)^b^ | 0.9011 (0.8728, 0.9294)^b^ |

Abbreviations: ACE, Abundance-based Coverage Estimator; API, apiaceous vegetables; DSS, dextran sulfate sodium; TWD, Total Western Diet.

Supplementary Table 10. Genus level differences between no DSS- and DSS-treated groups on Day 17. ^#^Positive and negative differences indicate the increased and decreased abundance after DSS treatment, respectively.

| Taxon | Diet | +DSS mean and 95% CI | -DSS mean and 95% CI | +DSS versus -DSS difference^#^ | T -statistic | Adjusted p-value |
| --- | --- | --- | --- | --- | --- | --- |
| Lachnospiraceae_UCG_001 | TWD + 42% API | -1.615  (-2.844, -0.387) | 0.689  (-0.114, 1.493) | -2.305 | -3.116 | 0.011 |
| uncultured | TWD + 42% API | 4.38  (3.903, 4.858) | 3.509  (3.2, 3.818) | 0.872 | 3.042 | 0.013 |
| Clostridia_UCG_014 | TWD + 42% API | -1.564  (-2.622, -0.506) | 1.42  (0.736, 2.104) | -2.984 | -4.701 | < 0.001 |
| Clostridia_vadinBB60_group | TWD + 21% API | 1.924  (1.124, 2.723) | -0.298  (-0.904, 0.309) | 2.221 | 4.393 | < 0.001 |
| Acetatifactor | TWD + 42% API | 0.981  (-0.032, 1.994) | -0.859  (-1.514, -0.204) | 1.84 | 3.027 | 0.013 |
| Pseudomonas | TWD + 42% API | -1.558  (-1.892, -1.224) | -0.871  (-1.089, -0.652) | -0.688 | -3.42 | 0.005 |
| Lachnospiraceae_NK4A136_group | TWD | 0.399  (-0.147, 0.944) | 2.269  (1.81, 2.728) | -1.87 | -5.207 | < 0.001 |
| Lactobacillus | TWD + 42% API | -0.863  (-2.091, 0.365) | 3.55  (2.728, 4.372) | -4.413 | -5.928 | < 0.001 |
| Roseburia | TWD | -1.435  (-1.933, -0.936) | 2.374  (1.955, 2.794) | -3.809 | -11.597 | < 0.001 |
| Roseburia | TWD + 21% API | -1.664  (-2.223, -1.105) | 2.295  (1.875, 2.714) | -3.958 | -11.238 | < 0.001 |
| Roseburia | TWD + 42% API | -1.595  (-2.243, -0.948) | 2.213  (1.793, 2.632) | -3.808 | -9.799 | < 0.001 |
| Bacteroides | TWD | 5.42  (4.842, 5.998) | 3.32  (2.83, 3.81) | 2.1 | 5.501 | < 0.001 |
| Bacteroides | TWD + 21% API | 5.296  (4.651, 5.942) | 2.314  (1.824, 2.804) | 2.982 | 7.303 | < 0.001 |
| Bacteroides | TWD + 42% API | 4.407  (3.662, 5.152) | 1.79  (1.3, 2.279) | 2.617 | 5.827 | < 0.001 |
| Christensenellaceae_R_7_group | TWD | -1.427  (-1.653, -1.2) | -0.624  (-0.815, -0.433) | -0.802 | -5.381 | < 0.001 |
| Erysipelatoclostridiaceae | TWD + 21% API | -1.657  (-2.342, -0.972) | -0.544  (-1.057, -0.031) | -1.113 | -2.58 | 0.04 |
| Escherichia_Shigella | TWD | -1.424 (-1.555, -1.293) | -1.721 (-1.831, -1.611) | 0.297 | 3.448 | 0.004 |
| Escherichia_Shigella | TWD + 42% API | -1.564  (-1.734, -1.394) | -1.852  (-1.961, -1.742) | 0.287 | 2.816 | 0.023 |
| Intestinimonas | TWD + 21% API | 1.806  (1.01, 2.603) | 0.32  (-0.276, 0.916) | 1.486 | 2.964 | 0.016 |
| Enterococcus | TWD | 2.12  (1.588, 2.652) | -1.424  (-1.871, -0.977) | 3.544 | 10.125 | < 0.001 |
| Romboutsia | TWD + 21% API | 3.435  (2.267, 4.603) | 0.575  (-0.301, 1.451) | 2.86 | 3.887 | 0.001 |
| Methylobacterium_Methylorubrum | TWD | -1.286  (-1.462, -1.109) | -1.721  (-1.869, -1.573) | 0.435 | 3.756 | 0.002 |
| Methylobacterium_Methylorubrum | TWD + 21% API | -1.482  (-1.68, -1.285) | -1.794  (-1.942, -1.646) | 0.311 | 2.503 | 0.048 |
| Methylobacterium_Methylorubrum | TWD + 42% API | -0.967  (-1.196, -0.738) | -1.852  (-1.999, -1.704) | 0.884 | 6.441 | < 0.001 |
| Colidextribacter | TWD + 21% API | 3.448  (3.043, 3.852) | 2.319  (2.016, 2.622) | 1.129 | 4.433 | < 0.001 |
| Blautia | TWD + 21% API | 4.136  (3.367, 4.904) | 2.179  (1.599, 2.759) | 1.957 | 4.031 | < 0.001 |
| Blautia | TWD + 42% API | 4.038  (3.15, 4.927) | 2.385  (1.805, 2.966) | 1.653 | 3.092 | 0.012 |
| ASF356 | TWD | 2.096  (1.158, 3.033) | -1.622  (-2.409, -0.835) | 3.718 | 6.027 | < 0.001 |
| Oscillibacter | TWD + 21% API | 3.77  (3.312, 4.228) | 2.164  (1.821, 2.508) | 1.605 | 5.565 | < 0.001 |
| A2 | TWD | -1.424  (-2.03, -0.818) | 2.874  (2.365, 3.383) | -4.298 | -10.775 | < 0.001 |
| A2 | TWD + 21% API | -1.656  (-2.335, -0.976) | 2.481  (1.972, 2.99) | -4.137 | -9.665 | < 0.001 |
| A2 | TWD + 42% API | -1.564  (-2.352, -0.777) | 2.296  (1.787, 2.805) | -3.86 | -8.169 | < 0.001 |
| Tuzzerella | TWD | -0.314  (-0.878, 0.25) | 0.977  (0.502, 1.453) | -1.291 | -3.475 | 0.004 |
| Tuzzerella | TWD + 21% API | 1.643  (1.011, 2.274) | 0.566  (0.09, 1.041) | 1.077 | 2.705 | 0.03 |
| Akkermansia | TWD | 6.169  (5.764, 6.575) | 5.17  (4.829, 5.51) | 1 | 3.745 | 0.002 |
| Akkermansia | TWD + 42% API | 5.622  (5.095, 6.149) | 4.743  (4.402, 5.084) | 0.879 | 2.781 | 0.025 |
| Erysipelatoclostridium | TWD | 2.961  (2.255, 3.668) | 0.993  (0.4, 1.586) | 1.969 | 4.236 | < 0.001 |
| Erysipelatoclostridium | TWD + 21% API | 3.176  (2.384, 3.968) | 0.262  (-0.331, 0.855) | 2.914 | 5.844 | < 0.001 |
| Eubacterium_xylanophilum_group | TWD | -1.39  (-1.982, -0.798) | 0.046  (-0.456, 0.548) | -1.436 | -3.672 | 0.002 |
| Eubacterium_xylanophilum_group | TWD + 21% API | -1.641  (-2.302, -0.98) | 2.149  (1.647, 2.651) | -3.79 | -9.061 | < 0.001 |
| Eubacterium_xylanophilum_group | TWD + 42% API | -1.5  (-2.263, -0.738) | 2.777  (2.275, 3.279) | -4.277 | -9.297 | < 0.001 |
| UBA1819 | TWD | -1.147  (-1.256, -1.038) | -1.721  (-1.812, -1.63) | 0.574 | 8.015 | < 0.001 |
| UBA1819 | TWD + 42% API | -1.564  (-1.706, -1.423) | -1.852  (-1.943, -1.76) | 0.287 | 3.385 | 0.005 |
| Coriobacteriaceae_UCG_002 | TWD + 21% API | 2.815  (1.466, 4.165) | 5.518  (4.488, 6.549) | -2.703 | -3.16 | 0.01 |
| Paraclostridium | TWD | 5.052  (4.853, 5.25) | -1.721  (-1.887, -1.554) | 6.773 | 51.907 | < 0.001 |
| Eubacterium_coprostanoligenes_group | TWD | 0.819  (-0.027, 1.664) | 3.031  (2.321, 3.742) | -2.213 | -3.975 | < 0.001 |
| UCG_009 | TWD | -0.036  (-0.56, 0.488) | -1.424  (-1.864, -0.984) | 1.388 | 4.023 | < 0.001 |
| UCG_009 | TWD + 21% API | 0.172  (-0.416, 0.759) | -1.637  (-2.077, -1.196) | 1.808 | 4.886 | < 0.001 |
| Muribaculaceae | TWD | -1.406  (-1.812, -1) | 2.637  (2.296, 2.979) | -4.043 | -15.126 | < 0.001 |
| Eubacterium_nodatum_group | TWD | 1.401  (0.778, 2.023) | 0.363  (-0.16, 0.885) | 1.038 | 2.534 | 0.045 |
| Eubacterium_nodatum_group | TWD + 21% API | -1.482  (-2.18, -0.784) | -0.253  (-0.775, 0.27) | -1.23 | -2.799 | 0.024 |
| Anaerotruncus | TWD | -1.425  (-2.038, -0.813) | -0.158  (-0.673, 0.357) | -1.267 | -3.142 | 0.01 |
| Turicibacter | TWD | 1.042  (0.315, 1.769) | 3.455  (2.841, 4.068) | -2.413 | -5.031 | < 0.001 |
| UCG_010 | TWD | -0.883  (-1.412, -0.353) | 0.396  (-0.049, 0.84) | -1.278 | -3.669 | 0.002 |
| Eubacterium_fissicatena_group | TWD | -1.428  (-2.153, -0.704) | 3.148  (2.532, 3.764) | -4.576 | -9.555 | < 0.001 |
| Eubacterium_fissicatena_group | TWD + 21% API | -1.653  (-2.46, -0.845) | 1.879  (1.263, 2.495) | -3.532 | -6.902 | < 0.001 |
| Lachnospiraceae_UCG_004 | TWD | -1.424  (-1.971, -0.877) | -0.28  (-0.739, 0.179) | -1.144 | -3.18 | 0.01 |
| Lachnospiraceae_UCG_004 | TWD + 21% API | -1.656  (-2.269, -1.043) | -0.383  (-0.842, 0.076) | -1.273 | -3.297 | 0.007 |
| Staphylococcus | TWD + 21% API | -0.741  (-1.234, -0.248) | -1.695  (-2.064, -1.325) | 0.954 | 3.073 | 0.012 |
| Dorea | TWD | -1.441  (-1.683, -1.199) | -1.011  (-1.216, -0.806) | -0.43 | -2.692 | 0.03 |
| Peptococcus | TWD | -1.424  (-1.586, -1.262) | -1.721  (-1.857, -1.585) | 0.297 | 2.782 | 0.025 |
| Parvibacter | TWD + 42% API | -1.564  (-2.188, -0.94) | -0.443  (-0.846, -0.039) | -1.122 | -2.995 | 0.014 |
| Parasutterella | TWD | 4.167  (3.5, 4.834) | 1.485  (0.925, 2.045) | 2.682 | 6.112 | < 0.001 |
| Parasutterella | TWD + 21% API | 3.198  (2.45, 3.946) | 0.638  (0.078, 1.198) | 2.56 | 5.436 | < 0.001 |
| Parasutterella | TWD + 42% API | 3.565  (2.699, 4.432) | 0.348  (-0.212, 0.908) | 3.217 | 6.189 | < 0.001 |
| Pantoea | TWD | -1.426  (-1.59, -1.261) | -1.721  (-1.86, -1.582) | 0.295 | 2.721 | 0.029 |

Abbreviations: API, apiaceous vegetables; DSS, dextran sulfate sodium; TWD, Total Western Diet.

Supplementary Table 11. Genus level differences in mice fed TWD supplemented with 21% or 42% API and no DSS treatment on Day 17. Significantly different group comparisons at genera taxonomy level are shown. Positive and negative values in the estimated difference column indicate higher and lower abundance in the group listed first.

| Taxon | Contrast | Estimated difference | T-statistic | Adjusted p-value |
| --- | --- | --- | --- | --- |
| Acetatifactor | (TWD + 21% API) - TWD | 1.532 | 3.283 | 0.014 |
| Bacteroides | (TWD + 21% API) - TWD | -1.006 | -2.884 | 0.036 |
| Bacteroides | (TWD + 42% API) - TWD | -1.531 | -4.387 | < 0.001 |
| Christensenellaceae_R_7_group | (TWD + 21% API) - TWD | -0.815 | -5.992 | < 0.001 |
| Christensenellaceae_R_7_group | (TWD + 42% API) - TWD | -1.227 | -9.03 | < 0.001 |
| Christensenellaceae_R_7_group | (TWD + 42% API) - (TWD + 21% API) | -0.413 | -3.037 | 0.024 |
| Clostridia_UCG_014 | (TWD + 42% API) - TWD | 1.742 | 3.574 | 0.006 |
| Dorea | (TWD + 21% API) - TWD | -0.783 | -5.366 | < 0.001 |
| Dorea | (TWD + 42% API) - TWD | -0.841 | -5.762 | < 0.001 |
| Eubacterium_coprostanoligenes_group | (TWD + 21% API) - TWD | -1.64 | -3.237 | 0.016 |
| Eubacterium_fissicatena_group | (TWD + 21% API) - TWD | -1.269 | -2.893 | 0.036 |
| Eubacterium_fissicatena_group | (TWD + 42% API) - TWD | -5 | -11.397 | < 0.001 |
| Eubacterium_fissicatena_group | (TWD + 42% API) - (TWD + 21% API) | -3.73 | -8.504 | < 0.001 |
| Eubacterium_xylanophilum_group | (TWD + 21% API) - TWD | 2.103 | 5.882 | < 0.001 |
| Eubacterium_xylanophilum_group | (TWD + 42% API) - TWD | 2.731 | 7.638 | < 0.001 |
| Holdemania | (TWD + 42% API) - TWD | -0.288 | -3.164 | 0.018 |
| Lachnospiraceae_FCS020_group | (TWD + 21% API) - TWD | 2.303 | 4.999 | < 0.001 |
| Lachnospiraceae_FCS020_group | (TWD + 42% API) - TWD | 2.485 | 5.395 | < 0.001 |
| Lachnospiraceae_NK4A136_group | (TWD + 21% API) - TWD | 1.594 | 4.874 | < 0.001 |
| Lachnospiraceae_NK4A136_group | (TWD + 42% API) - TWD | 1.017 | 3.109 | 0.02 |
| Lachnospiraceae_UCG_001 | (TWD + 42% API) - TWD | 2.41 | 4.211 | < 0.001 |
| Muribaculaceae | (TWD + 21% API) - TWD | -0.988 | -4.057 | 0.001 |
| Muribaculaceae | (TWD + 42% API) - TWD | -2.272 | -9.332 | < 0.001 |
| Muribaculaceae | (TWD + 42% API) - (TWD + 21% API) | -1.284 | -5.275 | < 0.001 |
| Parasutterella | (TWD + 42% API) - TWD | -1.136 | -2.847 | 0.038 |
| Pseudomonas | (TWD + 42% API) - TWD | 0.85 | 5.458 | < 0.001 |
| Pseudomonas | (TWD + 42% API) - (TWD + 21% API) | 0.667 | 4.282 | < 0.001 |
| Romboutsia | (TWD + 21% API) - TWD | -2.363 | -3.785 | 0.003 |
| Romboutsia | (TWD + 42% API) - TWD | -1.953 | -3.128 | 0.02 |
| Ruminococcaceae | (TWD + 21% API) - TWD | 1.571 | 3.198 | 0.017 |
| Ruminococcus | (TWD + 21% API) - TWD | 1.891 | 3.92 | 0.002 |
| Ruminococcus | (TWD + 42% API) - TWD | 3.185 | 6.601 | < 0.001 |

Abbreviations: API, apiaceous vegetables; DSS, dextran sulfate sodium; TWD, Total Western Diet.

Supplementary Table 12. Genus level differences in mice fed TWD supplemented with 21% or 42% API and challenged with DSS on Day 17. Significantly different group comparisons at genera taxonomy level are shown. Positive and negative values in the estimated difference column indicate higher and lower abundance in the group listed first.

| Taxon | Contrast | Estimated difference | T-statistic | Adjusted  p-value |
| --- | --- | --- | --- | --- |
| Acetatifactor | (TWD + 42% API) - TWD | 1.908 | 2.961 | 0.026 |
| Adlercreutzia | (TWD + 21% API) - TWD | -0.867 | -3.46 | 0.008 |
| Adlercreutzia | (TWD + 42% API) - TWD | -0.776 | -2.838 | 0.036 |
| Blautia | (TWD + 21% API) - TWD | 1.695 | 3.262 | 0.013 |
| Blautia | (TWD + 42% API) - TWD | 1.598 | 2.823 | 0.036 |
| Clostridia_UCG_014 | (TWD + 21% API) - TWD | 1.999 | 3.241 | 0.013 |
| Clostridia_UCG_014 | (TWD + 42% API) - (TWD + 21% API) | -2.6 | -3.691 | 0.004 |
| Clostridia_vadinBB60_group | (TWD + 21% API) - TWD | 2.848 | 5.265 | < 0.001 |
| Clostridia_vadinBB60_group | (TWD + 42% API) - TWD | 1.871 | 3.179 | 0.015 |
| Colidextribacter | (TWD + 21% API) - TWD | 0.918 | 3.36 | 0.01 |
| Coriobacteriaceae_UCG_002 | (TWD + 42% API) - (TWD + 21% API) | 2.862 | 2.759 | 0.043 |
| Enterococcus | (TWD + 21% API) - TWD | -3.099 | -7.694 | < 0.001 |
| Enterococcus | (TWD + 42% API) - TWD | -3.222 | -7.334 | < 0.001 |
| Eubacterium_nodatum_group | (TWD + 21% API) - TWD | -2.883 | -6.118 | < 0.001 |
| Eubacterium_nodatum_group | (TWD + 42% API) - TWD | -1.831 | -3.562 | 0.006 |
| Lachnospiraceae_FCS020_group | (TWD + 21% API) - TWD | 1.89 | 3.242 | 0.013 |
| Lachnospiraceae_FCS020_group | (TWD + 42% API) - TWD | 2.927 | 4.603 | < 0.001 |
| Lachnospiraceae_NK4A136_group | (TWD + 21% API) - TWD | 2.69 | 6.515 | < 0.001 |
| Lachnospiraceae_NK4A136_group | (TWD + 42% API) - TWD | 3.008 | 6.681 | < 0.001 |
| Lactobacillus | (TWD + 21% API) - TWD | -2.295 | -3.167 | 0.015 |
| Lactobacillus | (TWD + 42% API) - TWD | -3.91 | -4.975 | < 0.001 |
| Methylobacterium_Methylorubrum | (TWD + 42% API) - (TWD + 21% API) | 0.515 | 3.382 | 0.01 |
| Muribaculaceae | (TWD + 21% API) - TWD | 2.713 | 8.832 | < 0.001 |
| Muribaculaceae | (TWD + 42% API) - TWD | 2.293 | 6.847 | < 0.001 |
| Oscillibacter | (TWD + 21% API) - TWD | 1.622 | 5.243 | < 0.001 |
| Oscillibacter | (TWD + 42% API) - TWD | 1.002 | 2.969 | 0.026 |
| Paraclostridium | (TWD + 21% API) - TWD | -6.707 | -44.676 | < 0.001 |
| Paraclostridium | (TWD + 42% API) - TWD | -6.616 | -40.391 | < 0.001 |
| Parvibacter | (TWD + 42% API) - (TWD + 21% API) | -1.464 | -3.524 | 0.007 |
| Ruminococcus | (TWD + 42% API) - TWD | 1.848 | 2.851 | 0.036 |
| Turicibacter | (TWD + 21% API) - TWD | 2.71 | 4.928 | < 0.001 |
| Turicibacter | (TWD + 42% API) - TWD | 3.093 | 5.163 | < 0.001 |
| Tuzzerella | (TWD + 21% API) - TWD | 1.957 | 4.587 | < 0.001 |
| UBA1819 | (TWD + 21% API) - TWD | -0.509 | -6.172 | < 0.001 |
| UBA1819 | (TWD + 42% API) - TWD | -0.417 | -4.642 | < 0.001 |
| uncultured | (TWD + 21% API) - TWD | 0.929 | 3.338 | 0.01 |
| uncultured | (TWD + 42% API) - TWD | 1.038 | 3.419 | 0.009 |

Abbreviations: API, apiaceous vegetables; DSS, dextran sulfate sodium; TWD, Total Western Diet.

Supplementary Table 13. Selected metabolites from untargeted metabolomics analysis. Significantly up- and down-regulated metabolites on Day 12 after consumption of TWD supplemented with or without API. Data are presented as mean ± S.D. (arbitrary unit). ^***^*p*<0.001 between D0 and D12 in each diet group.

|  | D0 | | | |  | | D12 | | | |
| --- | --- | --- | --- | --- | --- | --- | --- | --- | --- | --- |
| Metabolites | TWD | TWD+21%API | TWD+42%API |  | | TWD | | TWD+21%API | TWD+42%API |  |
| Arachidonic acid (C20:4) | 28.7±10.0 | 46.3±20.6 | 39.3±14.9 |  | | 106.2±21.3^***^ | | 119.1±23.5^***^ | 109.1±35.5^***^ |  |
| Acetaldehyde | 80±43.2 | 61.5±33.2 | 95.8±75.9 |  | | 167.1±40.2^***^ | | 205.1±35.9^***^ | 237.2±45.5^***^ |  |
| Acrolein | 9.8±5.8 | 8.3±2.0 | 12.5±10.4 |  | | 39.6±16.2^***^ | | 50.6±12.6^***^ | 49.1±9.2^***^ |  |
| Butanal | 77.3±76.6 | 68.5±55.2 | 119.9±127.7 |  | | 274.8±80.6^***^ | | 299.7±79.8^***^ | 269.2±43.2^***^ |  |
| Pentanal | 232.4±197.0 | 203.5±165.7 | 321.6±313.4 |  | | 539.9±155.0^***^ | | 603.1±178.0^***^ | 580.7±94.6^***^ |  |
| Pentadecylic acid (C15:0) | 84.1±44.1 | 65.5±40.4 | 86.8±49.6 |  | | 159.6±45.6^***^ | | 191±58.9^***^ | 178±59.4^***^ |  |
| Palmitoleic acid (C16:1) | 63.1±8.9 | 74.8±10.8 | 72.1±18.8 |  | | 24.3±6.0^***^ | | 28.1±4.6^***^ | 28.1±5.9^***^ |  |
| α-Linolenic acid (C18:3) | 106.8±36.0 | 164.6±34.2 | 136.1±56.0 |  | | 12.6±6.1^***^ | | 20.5±7.7^***^ | 26.1±13.1^***^ |  |
| Linoleic acid (C18:2) | 678.9±97.6 | 821.6±88.1 | 756.6±172.5 |  | | 257.1±60.8^***^ | | 358.6±83.4^***^ | 394.1±112.1^***^ |  |
| Oleic acid (C18:1) | 442.5±41.5 | 468.6±66.4 | 483.3±113.3 |  | | 284.7±40.4^***^ | | 313.9±58.2^***^ | 316.7±52.0^***^ |  |
| Eicosapentaenoic acid (C20:5) | 34±12.5 | 57.2±16.1 | 49.2±20.9 |  | | 5.1±1.5^***^ | | 5.9±1.3^***^ | 4.3±1.6^***^ |  |
| Acetic acid (C2:0) | 411.7±69.3 | 336.8±63.9 | 339.6±76.6 |  | | 187±50.3^***^ | | 176±26.5^***^ | 216.4±40.8^***^ |  |
| Butyric acid (C4:0) | 328.3±109.9 | 258.4±108.9 | 258.4±134.3 |  | | 147.8±52.6^***^ | | 155.5±41.4^***^ | 180.2±63.4^***^ |  |

Abbreviations: API, apiaceous vegetables; TWD, Total Western Diet.
